# Prospective, randomized, controlled trial assessing the effects of a driving pressure–limiting strategy for patients with acute respiratory distress syndrome due to community-acquired pneumonia (STAMINA trial): protocol and statistical analysis plan

**DOI:** 10.62675/2965-2774.20240210-en

**Published:** 2024-04-26

**Authors:** STAMINA Study Group Investigators, Israel Silva Maia, Fernando Azevedo Medrado, Lucas Tramujas, Bruno Martins Tomazini, Júlia Souza Oliveira, Erica Regina Ribeiro Sady, Letícia Galvão Barbante, Marina Lazzari Nicola, Rodrigo Magalhães Gurgel, Lucas Petri Damiani, Karina Leal Negrelli, Tamiris Abait Miranda, Eliana Santucci, Nanci Valeis, Ligia Nasi Laranjeira, Glauco Adrieno Westphal, Ruthy Perotto Fernandes, Cássio Luis Zandonai, Mariangela Pimentel Pincelli, Rodrigo Cruvinel Figueiredo, Cíntia Loss Sartori Bustamante, Luiz Fernando Norbin, Emerson Boschi, Rafael Lessa, Marcelo Pereira Romano, Mieko Cláudia Miura, Meton Soares de Alencar, Vicente Cés de Souza Dantas, Priscilla Alves Barreto, Mauro Esteves Hernandes, Cintia Magalhães Carvalho Grion, Alexandre Sanches Laranjeira, Ana Luiza Mezzaroba, Marina Bahl, Ana Carolina Starke, Rodrigo Santos Biondi, Felipe Dal-Pizzol, Eliana Bernadete Caser, Marlus Muri Thompson, Andrea Allegrini Padial, Viviane Cordeiro Veiga, Rodrigo Thot Leite, Gustavo Araújo, Mário Guimarães, Priscilla de Aquino Martins, Fábio Holanda Lacerda, Conrado Roberto Hoffmann, Livia Melro, Eduardo Pacheco, Gustavo Adolfo Ospina-Táscon, Juliana Carvalho Ferreira, Fabricio Jocundo Calado Freires, Flávia Ribeiro Machado, Alexandre Biasi Cavalcanti, Fernando Godinho Zampieri

**Affiliations:** 1 Hcor-Hospital do Coração Research Institute São Paulo SP Brazil Research Institute, Hcor-Hospital do Coração - São Paulo (SP), Brazil.; 2 Universidade de São Paulo Department of Anesthesiology, Pain, and Intensive Care São Paulo SP Brazil Department of Anesthesiology, Pain, and Intensive Care, Universidade de São Paulo - São Paulo (SP), Brazil.; 3 Brazilian Research in Intensive Care Network São Paulo SP Brazil Brazilian Research in Intensive Care Network (BRICNet) - São Paulo (SP), Brazil.; 4 Centro Hospitalar Unimed Joinville Joinville SC Brazil Centro Hospitalar Unimed Joinville - Joinville (SC), Brazil.; 5 Hospital Nereu Ramos Florianópolis SC Brazil Hospital Nereu Ramos - Florianópolis (SC), Brazil.; 6 Hospital e Maternidade São José Colatina ES Brazil Hospital e Maternidade São José - Colatina (ES), Brazil.; 7 Linhares Medical Center Linhares ES Brazil Linhares Medical Center - Linhares (ES), Brazil.; 8 Hospital Geral de Caxias do Sul Caxias do Sul RS Brazil Hospital Geral de Caxias do Sul - Caxias do Sul (RS), Brazil.; 9 Hcor-Hospital do Coração São Paulo SP Brazil Hcor-Hospital do Coração - São Paulo (SP), Brazil.; 10 Hospital São Vicente de Paulo Barbalha CE Brazil Hospital São Vicente de Paulo - Barbalha (CE), Brazil.; 11 Hospital Marcílio Dias Rio de Janeiro RJ Brazil Hospital Marcílio Dias - Rio de Janeiro (RJ), Brazil.; 12 Santa Casa de Votuporanga Votuporanga SP Brazil Santa Casa de Votuporanga - Votuporanga (SP), Brazil.; 13 Universidade Estadual de Londrina Hospital Universitário Londrina PR Brazil Hospital Universitário, Universidade Estadual de Londrina - Londrina (PR), Brazil.; 14 Hospital Araucária de Londrina Londrina PR Brazil Hospital Araucária de Londrina - Londrina (PR), Brazil.; 15 Universidade Federal de Santa Catarina Hospital Universitário Florianópolis SC Brazil Hospital Universitário, Universidade Federal de Santa Catarina - Florianópolis (SC), Brazil.; 16 Hospital Brasília Brasília DF Brazil Hospital Brasília - Brasília (DF), Brazil.; 17 Hospital São José Criciúma SC Brazil Hospital São José - Criciúma (SC), Brazil.; 18 Hospital Unimed Vitória Vitória SC Brazil Hospital Unimed Vitória - Vitória (SC), Brazil.; 19 Hospital Evangélico de Cachoeiro de Itapemirim Cachoeiro de Itapemirim ES Brazil Hospital Evangélico de Cachoeiro de Itapemirim - Cachoeiro de Itapemirim (ES), Brazil.; 20 Instituto Baía Sul Florianópolis SC Brazil Instituto Baía Sul - Florianópolis (SC), Brazil.; 21 BP - A Beneficência Portuguesa de São Paulo São Paulo SP Brazil BP - A Beneficência Portuguesa de São Paulo - São Paulo (SP), Brazil.; 22 Imperial Hospital de Caridade Florianópolis SC Brazil Imperial Hospital de Caridade - Florianópolis (SC), Brazil.; 23 Santa Casa de Misericórdia de Barretos Barretos SP Brazil Santa Casa de Misericórdia de Barretos - Barretos (SP), Brazil.; 24 Hospital Estadual Dr. Jayme Santos Neves Serra ES Brazil Hospital Estadual Dr. Jayme Santos Neves - Serra (ES), Brazil.; 25 Hospital Otoclínica Fortaleza CE Brazil Hospital Otoclínica - Fortaleza (CE), Brazil.; 26 Hospital Regional Hans Dieter Schmidt Joinville SC Brazil Hospital Regional Hans Dieter Schmidt - Joinville (SC), Brazil.; 27 Hospital Samaritano São Paulo SP Brazil Hospital Samaritano, São Paulo (SP), Brazil.; 28 Hospital Sepaco São Paulo SP Brazil Hospital Sepaco - São Paulo (SP), Brazil.; 29 Universidad ICESI Fundación Valle del Lili Colombia CO Fundación Valle del Lili - Universidad ICESI - Colombia, CO.; 30 Universidade de São Paulo Hospital das Clínicas Department of Pneumology São Paulo SP Brazil Department of Pneumology, Instituto do Coração, Hospital das Clínicas, Faculdade de Medicina, Universidade de São Paulo - São Paulo (SP), Brazil.; 31 Universidade Federal de São Paulo Department of Anesthesiology, Pain, and Intensive Care São Paulo SP Brazil Department of Anesthesiology, Pain, and Intensive Care, Universidade Federal de São Paulo - São Paulo (SP), Brazil.; 32 University of Alberta and Alberta Health Services - Edmonton Faculty of Medicine and Dentistry Department of Critical Care Medicine Alberta Canada Department of Critical Care Medicine, Faculty of Medicine and Dentistry, University of Alberta and Alberta Health Services - Edmonton, Alberta, Canada.

**Keywords:** Extracorporeal membrane oxygenation, Respiratory distress syndrome, Positive pressure respiration, Respiration, artificial, Ventilator-induced lung injury, Pneumonia

## Abstract

**Background:**

Driving pressure has been suggested to be the main driver of ventilator-induced lung injury and mortality in observational studies of acute respiratory distress syndrome. Whether a driving pressure-limiting strategy can improve clinical outcomes is unclear.

**Objective:**

To describe the protocol and statistical analysis plan that will be used to test whether a driving pressure-limiting strategy including positive end-expiratory pressure titration according to the best respiratory compliance and reduction in tidal volume is superior to a standard strategy involving the use of the ARDSNet low-positive end-expiratory pressure table in terms of increasing the number of ventilator-free days in patients with acute respiratory distress syndrome due to community-acquired pneumonia.

**Methods:**

The ventilator STrAtegy for coMmunIty acquired pNeumoniA (STAMINA) study is a randomized, multicenter, open-label trial that compares a driving pressure-limiting strategy to the ARDSnet low-positive end-expiratory pressure table in patients with moderate-to-severe acute respiratory distress syndrome due to community-acquired pneumonia admitted to intensive care units. We expect to recruit 500 patients from 20 Brazilian and 2 Colombian intensive care units. They will be randomized to a driving pressure-limiting strategy group or to a standard strategy using the ARDSNet low-positive end-expiratory pressure table. In the driving pressure-limiting strategy group, positive end-expiratory pressure will be titrated according to the best respiratory system compliance.

**Outcomes:**

The primary outcome is the number of ventilator-free days within 28 days. The secondary outcomes are in-hospital and intensive care unit mortality and the need for rescue therapies such as extracorporeal life support, recruitment maneuvers and inhaled nitric oxide.

**Conclusion:**

STAMINA is designed to provide evidence on whether a driving pressure-limiting strategy is superior to the ARDSNet low-positive end-expiratory pressure table strategy for increasing the number of ventilator-free days within 28 days in patients with moderate-to-severe acute respiratory distress syndrome. Here, we describe the rationale, design and status of the trial.

## INTRODUCTION

Despite recent advances in the care and management of ventilated patients, mortality due to acute respiratory distress syndrome (ARDS) remains high.^(1)^ The severity of ARDS of respiratory etiology was especially evident during the coronavirus disease 2019 (COVID-19) pandemic, during which the mortality of ventilated patients exceeded 50%.^(2^^,^^3)^ Strategies that improve the care of these patients are necessary to decrease morbidity and mortality.

Cyclic stretch of the pulmonary parenchyma is a major contributor to ventilation-induced lung injury (VILI) and varies according to the reduced resting volume of aerated lung in ARDS patients,^(4)^ which in turn is strongly correlated with compliance of the respiratory system. Driving pressure (DP) is an easily measured variable that is equal to the tidal volume divided by the compliance of the respiratory system. Because of these properties, DP has been proposed to be a key mediator of VILI.^(5)^ The association between DP and mortality during mechanical ventilation is supported by observational data.^(4^^,^^6)^ Nevertheless, it is currently unknown whether a treatment strategy limiting DP would be superior to a conventional ventilation strategy in terms of clinical outcomes.^(7)^ Effective strategies to reduce DP include best positive end-expiratory pressure (PEEP) titration and lower tidal volume. The standard PEEP handling strategy involves the arbitrary use of PEEP according to the need for an inspired fraction of oxygen (FiO_2_; "PEEP table").^(8^^,^^9)^ Nonetheless, it can be based on the best respiratory system compliance, which is inversely correlated with DP.^(5)^ By gradually adjusting PEEP levels while monitoring for changes in compliance, clinicians can determine the optimal PEEP setting that minimizes DP.^(10^^,^^11)^ Additionally, reducing the tidal volume below the standard 6mL/kg can further decrease the DP. Implementing these measures in ARDS patients allows for a personalized approach to ventilator management, reducing DP and potentially mitigating VILI.

The objective of this study was to assess whether a mechanical ventilation strategy focused on reducing DP can lead to improved clinical outcomes compared to the ARDSNet low-PEEP table strategy in patients with moderate-to-severe acute respiratory distress syndrome secondary to community-acquired pneumonia (CAP). Community-acquired pneumonia is responsible for 50% of ARDS cases in some clinical trials.^(1^^,^^12)^ Including only CAP patients can improve the recruitment rate, especially during pandemics, and decrease the chance of finding heterogeneous treatment effects between ARDS etiologies.

This study is being conducted to evaluate the impact of this novel approach on key clinical outcomes, such as ventilator-free days and overall improvement in respiratory parameters. By comparing these outcomes between the two ventilation strategies, we aim to determine whether a DP-limiting approach can provide superior benefits regarding clinically important outcomes and potentially pave the way for optimized ventilatory management in ARDS patients.

## METHODS

### Study design

The STAMINA (ventilator STrAtegy for coMmunIty acquired pNeumoniA) study is a randomized, multicenter, open-label trial that will compare two ventilatory strategies: a DP limiting strategy versus a standard strategy (ARDSNet low PEEP table) in patients with moderate-to-severe ARDS secondary to community-acquired pneumonia (CAP) who are hospitalized in intensive care units (ICUs). Eligible patients will receive one of these strategies. The protocol of this study is reported according to the recommendations of the SPIRIT 2013 Statement.^(13)^

### Objectives and outcomes

To evaluate whether a DP-limiting strategy for titrating PEEP according to best compliance is superior in terms of increasing the number of ventilator-free days compared to the standard strategy using a low-PEEP table. The primary, secondary, exploratory and safety outcomes are shown in table 1.

**Table 1:** Outcomes

Outcomes	
Primary	Ventilator-free days within 28 days from randomization or until hospital discharge measured as follows:
	D = zero (if the patient dies within 28 days in the hospital or remains on respiratory support with MV ≥ 28 days)
	D = 28 - x (if the patient is released from the hospital in < 28 days, where x represents the number of days with MV during hospitalization)
	The number of days on MV will be counted as every day the patient spent at least 12 hours on MV. If there is an interruption of MV followed by restart within 48 hours (extubation failure), the entire period will be computed as a single period. For tracheostomized patients, the same criterion is valid. One day of ventilation is computed whenever the patient persists more than 12 hours ventilated
Secondary	1. In-hospital mortality within 90 days
	2. ICU mortality within 90 days
	3. Need for rescue therapies (extracorporeal circulation, recruitment maneuver outside the study protocol, inhaled nitric oxide) within 28 days
Exploratory	1. Oxygenation, measured by the PaO_2_/FiO_2_ ratio during the first 3 days
	2. Driving pressure during the first 3 days
	3. Ventilatory ratio, defined as PaCO_2_ multiplied by minute ventilation divided by 100 × predicted body weight in kilograms × 37.5, measured in the first 3 days
	4. Oxygenation index, defined by MAP × FiO_2_ × 100 ÷ PaO_2_, measured in the first 3 days
	5. Mechanical power, defined by energy transfer (0.098) x respiratory rate x tidal volume x [peak airway pressure – 0.5 x (plateau pressure – PEEP)], measured in the first 3 days
	6. ICU-free days in 28 days
	7. ICU length of stay
	8. Hospital length of stay
Safety outcomes	1. Occurrence of barotrauma (subcutaneous emphysema, pneumothorax, or pneumomediastinum)
	2. Other serious adverse events possibly related to MV within 28 days

MV - mechanical ventilation; ICU - intensive care unit; PaO_2_ - partial pressure of arterial oxygen; FiO_2_ - fraction of inspired oxygen; PaCO_2_ - partial pressure of arterial carbon dioxide; MAP - mean airway pressure; PEEP - positive end-expiration pressure.

### Sites and recruitment

At least 20 Brazilian and 2 Colombian ICUs. The expected recruitment rate is 1 patient/month/site. The planned trial duration is estimated at 2.5 years.

### Inclusion and exclusion criteria

**Figure 1:**
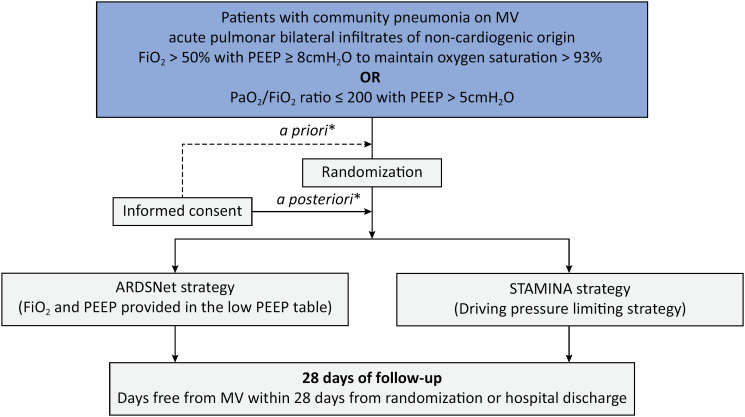
Study flow.

Patients must be ≥ 18 years old, with moderate or severe acute respiratory distress syndrome due to CAP and receiving mechanical ventilation. The eligibility criteria are detailed in table 2. The patient flowchart is detailed in figure 1.

**Table 2:** Eligibility criteria

Inclusion criteria	Exclusion criteria
Patients with community acquired pneumonia on invasive MV (pneumonia must have been the cause for initiation of mechanical ventilation) Acute bilateral infiltrate of nonexclusively cardiogenic origin, at the judgment of the attending physician One of the below: Inspired oxygen fraction above 50% with PEEP of at least 8cmH_2_O to maintain saturation above 93% OR PaO_2_/FiO_2_ < 200 with PEEP > 5cmH_2_O	Patients with inclusion criteria for more than 36 hours Intracranial hypertension or acute neurological disease (stroke, subarachnoid hemorrhage Refusal of the legal representative Patients under 18 years of age Patients considered not to be candidates for measures of full invasive support at the time of randomization, that is, patients who have some clear indication, at the time of randomization, for not instituting other invasive support measures Patient with bronchial fistula or barotrauma Patients with a history of home use of oxygen by chronic respiratory disease

MV - mechanical ventilation; PEEP - positive end-expiration pressure; PaO_2_ - partial pressure of arterial oxygen; FiO_2_ - fraction of inspired oxygen.

Note: Inclusion criterion 3a assumes the need for these parameters to maintain at least 93% oxygen saturation. If the patient is admitted with a high inspired fraction and/or high positive end-expiration pressure, it is recommended, if possible, to try to reduce ventilatory parameters (fraction of inspired oxygen and/or positive end-expiration pressure) to assess the oxygen saturation response before considering this criterion fulfilled.

MV - mechanical ventilation; FiO_2_ - fraction of inspired oxygen; PEEP - positive end-expiration pressure; PaO_2_ - partial pressure of arterial oxygen. * Informed consent can be signed by the next of kin before or after randomization.

### Study interventions

The study interventions will be performed from Day 0 (randomization) through Day 3. The ventilator procedures are summarized in table 3.

**Table 3:** Study interventions after randomization from D0 to D3

Variable	ARDSnet strategy	STAMINA strategy
Baseline adjustments		
Ventilator mode	Volume assist-control	Volume assist-control
Plateau pressure (cmH_2_O)	≤ 30	≤ 30
Initial tidal volume (mL/kg of predicted body weight)	6 or lower in order to maintain Pplat ≤ 30cmH_2_O	6 or lower in order to maintain Pplat ≤ 30cmH_2_O
Respiratory rate setting needed to achieve pH ≥ 7.2	Up to 35 breaths/minute	Up to 35 breaths/minute
Oxygenation goal	SpO_2_ 90 - 94% and PaO_2_ ≥ 60mmHg	SpO_2_ 90 - 94% and PaO_2_ ≥ 60mmHg
Flow wave format	Descending or squared	Descending or squared
Inspiratory pause	Maximum 0.5 seconds	Maximum 0.5 seconds
Flow (L/minute)	30 - 60	30 - 60
I:E	1:1 - 1:2	1:1 - 1:2
PEEP titration adjustments		
PEEP titration*	Low PEEP - FiO_2_ table	Incremental phase: up to PEEP of 20cmH_2_O Decremental phase: reduction in PEEP in steps of 2cmH_2_O PEEP titrated by best static respiratory system compliance
Peep titration stopping criteria		Peak airway pressure ≥ 40cmH_2_O Pplat pressure ≥ 33cmH_2_O Unstable cardiac arrythmias increase vasopressor dose > 20micg/minute†
Decremental phase		Inspiratory pause of 2 seconds at the end of each step to measure compliance value up to PEEP of 8cmH_2_O‡ Best PEEP will be the immediately previous value of a drop in compliance by at least 2mL/cmH_2_O in one decrease or by at least 1mL/cmH_2_O in two sequential decreases‡
Driving pressure	Not allowed to be controlled	Controlled to keep ≤ 14
Additional adjustments	If plateau pressure > 30cmH_2_O: – Reduction of tidal volume up to 4mL/kg of predicted body weight, followed by reduction in PEEP in steps of 2cmH_2_O (if not solved with reduction of TV) – If necessary, is permitted to increase RR up to 40 breaths/minute if pH < 7.2 and PaCO_2_ > 70mmHg with auto PEEP not greater than 2cmH_2_O	If DP > 14 and/or plateau pressure > 30: – Reduction of tidal volume up to 4mL/kg of predicted body weight, followed by reduction in PEEP in steps of 2cmH_2_O (if not solved with reduction of tidal volume) – If necessary, is permitted to increase RR up to 40 breaths/min if pH < 7.2 and PaCO_2_ > 70mmHg with auto PEEP not greater than 2cmH_2_O
Mechanics monitoring	Two times per day apart 8 hours from each assessment	Two times per day apart 8 hours from each assessment
Sedation	Deep sedation with RASS -5 and neuromuscular blockade in the first 72 hours if patient maintains in controlled ventilation. After this time, light sedation (RASS 0 to -2)	Deep sedation with RASS -5 and neuromuscular blockade in the first 72 hours if patient maintains in controlled ventilation. After this time, light sedation (RASS 0 to -2)
New titration procedure in less than 24 hours	Pplat > 30cmH_2_O, change body position (prone or supine), circuit depressurization	Pplat > 30cmH_2_O and/or DP > 14cmH_2_O, change body position (prone or supine), circuit depressurization
Titration routine	D0, D1, D2, D3 24 hours apart and any time if necessary	D0, D1, D2, D3 24 hours apart and anytime if necessary
PEEP weaning after 24 hours with PaO_2_/FiO_2_ ratio > 200	Decrease 2cmH_2_O at 8 hours intervals	Decrease 2cmH_2_O at 8 hours intervals
Ventilation weaning	FiO_2_ ≤ 50% with PEEP ≤ 10cmH_2_O	FiO2 ≤ 50% with PEEP ≤ 10cmH_2_O

Pplat - plateau pressure; SpO_2_ - oxygen saturation; PaO_2_ - partial pressure of arterial oxygen; I:E ratio - ratio of the duration of inspiration to the duration of expiration; PEEP - positive end-expiratory pressure; FiO_2_ - fraction of inspired oxygen; PaCO_2_ - partial pressure of arterial carbon dioxide; DP - driving pressure; RASS - Richmond Agitation-Sedation Scale; RR - respiratory rate.

The predicted weight should be calculated for all patients according to the following formula: men: predicted weight (kg) = 50 + 2.3 {[height (cm) x 0.394] - 60}, women: predicted weight (kg) = 45.5 + 2.3 {[height (cm) x 0.394] - 60}.

* In the STAMINA arm, the titration maneuver can be postponed on a given day if the patient has a PaO2/FiO2 ratio above 200, the plateau pressure is below 30cmH_2_O, the driving pressure is not higher than 14cmH_2_O and the positive end-expiratory pressure is not greater than 10cmH_2_O;

† if any stopping criterion rule is reached, the decremental phase will start from the positive end-expiratory pressure value where the incremental phase stops;

‡ if there is a tie between multiple positive end-expiratory pressure values, the lowest value of positive end-expiratory pressure will be used.

### Intervention group – STAMINA strategy

**Figure 2:**
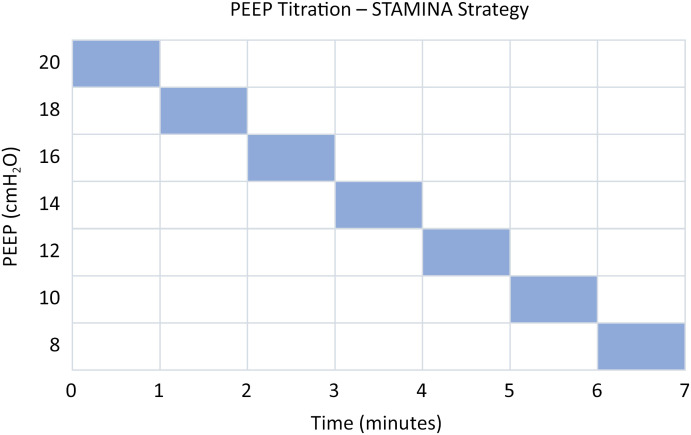
Positive end-expiratory pressure titration - STAMINA strategy.

The STAMINA strategy comprises a DP-limited strategy that includes PEEP titration guided by the best respiratory system compliance. PEEP is titrated as follows: initially, an incremental phase is performed, increasing PEEP up to 20cmH_2_O, respecting the safety stopping criteria detailed in table 3. Nevertheless, in the decremental phase, PEEP is reduced in 2cmH_2_O every one minute and maintained at the level with the best static compliance. Subsequently, the tidal volume and/or PEEP can be adjusted to maintain a DP equal to or lower than 14cmH_2_O and a plateau pressure (Pplat) equal or lower than 30cmH_2_O. A schematic is shown in figure 2.

This figure depicts the STAMINA strategy used to conduct positive end-expiratory pressure titration. The positive end-expiratory pressure was initially raised to 20cmH_2_O and subsequently decreased by 2cmH_2_O after 1 minute at each level until reaching 8cmH_2_O. Following this, the optimal positive end-expiratory pressure is determined based on the value that yields the best static compliance of the respiratory system.

PEEP - positive end-expiratory pressure.

### Control group – ARDSnet strategy

Positive end-expiratory pressure and fraction of inspired oxygen (FiO_2_) will be adjusted by the low-PEEP ARDSNet table (Table 4) to maintain oxygen saturation (SpO_2)_ between 90% and 94% or a partial pressure of arterial oxygen (PaO_2_) equal to or greater than 60mmHg. Subsequently, the tidal volume and/or PEEP can be adjusted to keep Pplat equal or lower than 30cmH_2_O, regardless of the DP.

**Table 4:** Low positive end-expiratory pressure-fraction of inspired oxygen table

FiO_2_ (%)	30	40	40	50	50	60	70	70	70	80	90	90	90	100
PEEP (cmH_2_O)	5	5	8	8	10	10	10	12	14	14	14	16	18	18 - 24

FiO_2_ - fraction of inspired oxygen; PEEP - positive end-expiratory pressure.

### Sample size

We plan to randomize 500 patients. This number of patients provides a power of at least 90% for detecting a difference of approximately 3 mechanical ventilation-free days and a 5% lower mortality rate in the treatment group.

Considering the raw data from the non-death-related part of the composite outcome (ventilator-free days) and a mortality rate of 60% in the Control group of the CoDEX study^(3)^ as a reference, our simulations reached an average of 4.7 mechanical ventilation-free days in 28 days for the Control group, with quartiles [3.7 - 5.8], standard deviation of 8.2, and a mean of 7.8 mechanical ventilation-free days for the Treatment group, with quartiles [6.5 - 9.2] and a standard deviation of 10.5.

**Figure 3:**
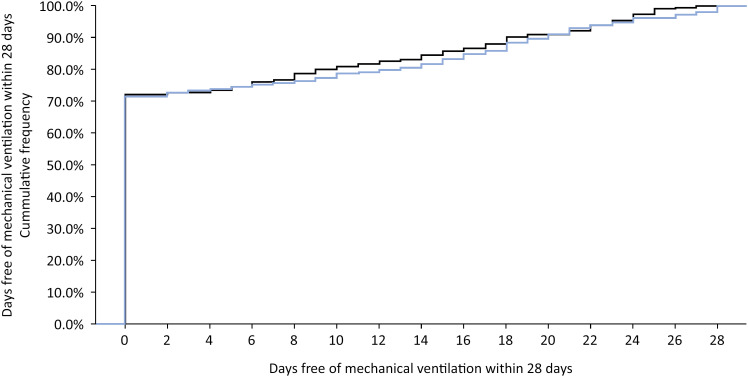
Cumulative distribution for the number of mechanical ventilator-free days within 28 days. Null scenario.

Power estimates were obtained from 2,000 simulations of different scenarios for the primary outcome, considering that death within 28 days would imply zero ventilator-free days, even if the patient was ventilator-free for at least one day during hospitalization. Figure 3 shows the cumulative distribution for the primary outcome from a simulation considering the null model (the effect in the Treatment group was identical to that in the Control group).

### Recruitment

Currently, patient enrollment is underway across 29 ICUs throughout Brazil and 2 in Colombia. These centers actively collaborate with the Brazilian Intensive Care Research Network (BRICNet), which has participated in previous studies, ensuring a high standard of patient recruitment and high-quality data. Most of these centers are located in the southern or southeastern region of Brazil, where we expect a higher incidence of community-acquired pneumonia related to higher population density associated with a lower temperature environment during fall and winter.

### Randomization and allocation concealment

The randomization list will be generated electronically using appropriate software. Randomization will be performed in blocks (variable block size) stratified by center and diagnosis of COVID-19. The confidentiality of the randomization list will be maintained through the central automated randomization system via the internet, which is available 24 hours a day (RedCap). The group in which the patient will be allocated will only be disclosed after the information is registered in the electronic system, which will prevent the investigator and the assistant team from predicting the treatment group to which the patient will be allocated. The investigator must visit the website used in the study (RedCap) to formally allocate the patients to the different treatment groups.

### Blinding

There will be no blinding due to the nature of the intervention, which will make it impossible to mask investigators, participants and outcome assessors. However, the outcome assessors only will have access to outcomes data after the end of the study.

### Data collection time sequence

**Figure 4:**
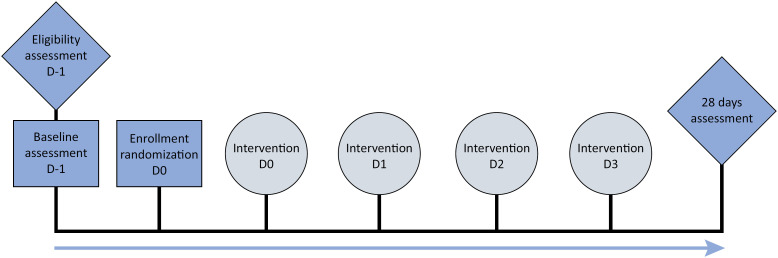
Participant timeline.

The data that will be collected are specified in table 5 according to figure 4.

**Table 5:** Data collection schedule

	D-1	D0	D1	D2	D3	D28	ICU discharge	Hospital discharge
Eligibility criteria	X							
Randomization		X						
Informed Consent	X							
Time between ICU admission and randomization		X						
Time of MV before randomization		X						
Date of birth		X						
Ventilatory parameters		X	X	X	X			
Arterial blood gas		X	X	X	X			
Sex at birth	X							
Height	X							
Comorbidities	X							
Pneumonia etiology	X							
Vasopressor dose		X	X	X	X	X	X	
RASS at randomization		X						
SAPS 3 parameters		X						
Protocol support: interventions		X	X	X	X			
Prone position		X	X	X	X		X	
Rescue therapies		X	X	X	X		X	
Vasopressors use		X	X	X	X		X	
Creatinine	X	X	X	X	X			
Total bilirubin	X	X	X	X	X			
Date and time of extubation			X	X	X	X	X	
Data and time of reintubation			X	X	X	X	X	
Vital status at ICU discharge						X	X	
Date of death						X	X	X
Number of days in use of MV in hospital						X	X	X
Date of tracheostomy						X	X	X
Data of ICU discharge							X	
Data of hospital discharge								X
Vital status on hospital discharge								X

ICU - intensive care unit; MV - mechanical ventilation; RASS - Richmond Agitation-Sedation Scale; SAPS 3 - Simplified Acute Physiology Score 3.

D - day.

### Data collection and management

We will abstract data routinely collected in the intensive care unit through a digital database with easy access via the internet (RedCap).

Several procedures will ensure the quality of the data, including the following:

1. All researchers will participate in a training session before the beginning of the study to ensure consistency of the study procedures, including data collection.
2. Researchers may call and/or attend brief online meetings with the Study Coordinating Center to resolve issues or problems that may arise, as well as participate in discussion groups by electronic messengers.
3. Data checks to identify inconsistencies will be conducted periodically. Centers will be notified of inconsistencies to provide corrections.
4. The Coordinating Center will review detailed monthly reports on the screening, inclusion, follow-up, consistency, and completeness of the data and will immediately take action to resolve any problems.
5. Site monitoring will be performed during the study for a sample of the sites. Such monitoring will be carried out remotely.

### Statistical analysis

A detailed statistical analysis plan can be found in the Appendix 2 in Supplementary Material. The summarized characteristics of the statistical analysis plan are described below. All analyses will adhere to the principle of a modified intention-to-treat approach. Exclusions of participants after randomization may occur due to refusals, given the retrospective (opt-out) nature of the informed consent. We will evaluate the effect of the STAMINA strategy versus the ARDSNet strategy on the primary outcome using a random ordinal model, with adjustment for baseline age, diagnosis of COVID-19, basal ventilatory ratio, and PEEP. The results will be reported as proportional odds ratio (ORp) with 95% confidence intervals. For binary secondary outcomes, we will compare odds ratios and 95% confidence intervals adjusted for the same variables and used random intercepts at the center. For continuous outcomes, we will present the mean difference and 95% confidence interval.

We will analyze the effect of interventions on the following interest subgroups:

1. Patients with and without a diagnosis of COVID-19 (confirmed by polymerase chain reaction - PCR);
2. Patients with DP above or below 15cmH_2_O prior to randomization.

### Data monitoring

The data monitoring at each site involved in this clinical trial will be carried out by a highly trained and qualified site management team. They will be responsible for conducting planned on-site visits to ensure data accuracy, integrity, and compliance with the study protocol and regulatory requirements. Moreover, the team will meticulously review source documents, case report forms, and other trial-related documents to verify the completeness and quality of the collected data.

### Adverse events

We will collect and review data on suspected unexpected severe adverse events (SAEs). Such events must be reported to the coordinating site within 24 hours. Any SAEs must be reported to the coordinating site within 24 hours. Any SAEs will be managed immediately by the treating team and will be adjudicated by the coordinator center team. Unexpected events will be reported to the research team within 24 hours. It is anticipated that the patient population in the ICU will experience a number of aberrations in laboratory values, signs and symptoms due to the severity of the underlying disease and the impact of standard treatments in the ICU. These events will not necessarily constitute adverse events or SAEs unless they are considered to be related to the study treatment or, in the principal investigator's clinical judgment, are not recognized events consistent with the participants underlying critical illness and/or chronic diseases and the expected clinical course. The main adverse event of interest is barotrauma (pneumothorax, pneumomediastinum, subcutaneous emphysema).

### Auditing

Auditing will be performed by the sponsor's operating team to help maintain objectivity and credibility in evaluating the trial's compliance with the protocol, regulatory requirements, and Good Clinical Practice (GCP) guidelines.

### Harms

Considering that the patient would receive mechanical ventilation support even if he was not participating in the study, we may expect a minimal increase in the risk already inherent in the context of mechanical ventilation, comprising the need for sedation, blood pressure drop (hypotension), increased risk of pneumonia and neuromuscular blocking agent use.

### Protocol violations and deviations

Major deviations relating to the inclusion criteria, exclusion criteria, study conduct and patient management must be reported to and monitored by the coordinating site.

### Trial organization and oversight

The steering committee of the trial has fourteen members and is responsible for developing the study protocol, drafting the manuscript and submitting the manuscript for publication (Appendix 1 - Supplementary Material). A team from the Hcor Research Institute is coordinating the study in association with the BRICNet. The Hcor Research Institute is responsible for conducting the study and for managing and controlling the quality of the data.

The data monitoring committee (DMC) is composed of an external statistician and two researchers who are all experts in critical care medicine (see DMC Charter in the Appendix 3 - Supplementary Material). The DMC is responsible for the interim analysis and for providing guidance to the steering committee regarding the continuation and safety of the trial after the interim analyses.

### Current trial status

Ethical approval was obtained on July 15, 2021. Since then, 207 patients have been randomly assigned to one of the study arms. The first patient was included in the study on September 4, 2021. The first DMC meeting was held on April 11, 2022. After analyzing the data according to the DMC Charter (Appendix 3 - Supplementary Material), the DMC recommended continuation of the study as planned in the protocol.

### Ethical consideration and dissemination

The trial was designed according to the guidelines for Good Clinical Practice and the principles of the Declaration of Helsinki. This study was approved by the Hcor Committee on Ethics in Research on July 15, 2021 (approval 4.848.945), and each participating center's ethics committee. Consent will be requested from all legal or family representatives. During the process of obtaining consent, the STAMINA study researchers will attempt to obtain the consent form before inclusion of the patient whenever possible. However, we believe that in several cases, obtaining informed consent after randomization would be fully justifiable given the urgency of the treatment decision. This study was approved by the Ethics Committee. Therefore, if it is not possible to contact the legal representative before inclusion in the study, consent will be obtained after inclusion, within 48 hours, through his or her family member/legal representative. The Informed Consent Form, on paper, should be prioritized. However, considering restrictions on family visits to hospital facilities, especially during the COVID-19 pandemic period, digital alternatives, such as e-mail or recorded phone calls, can be employed. If the patient dies before the investigator contacts the legal representative, the center's ethics committee will be informed of the data usage permissions. The study will be submitted for publication after completion irrespective of its findings. Manuscript preparation will be an inalienable responsibility of the steering committee. The main paper will be authored by the steering committee members plus the principal investigators of the top recruiting sites, who can contribute intellectually to the manuscript.

### Role of the sponsor source

Brazil's Ministry of Health—through its *Programa de Apoio e Desenvolvimento Institucional do Sistema Único de Saúde* (PROADI-SUS)—provides grant funding for STAMINA. The sponsor is not involved in the study design, data analysis, manuscript preparation or decision to submit the results for publication.

### Declaration of interest

The members of the Steering Committee declare no present disclosures.

## DISCUSSION

The STAMINA trial is planned as a randomized, multicenter, controlled clinical trial with the objective of comparing two ventilatory strategies in patients with ARDS due to CAP. The STAMINA strategy (intervention group) consists of limiting DP by optimizing PEEP guided by the best compliance of the respiratory system and by reducing tidal volume if the DP remains higher than 14cmH_2_O. In the ARDSnet group, PEEP titration will be performed using the low-PEEP-FiO_2_ table. Our aim is to assess whether this DP-limiting strategy will reduce the number of mechanical ventilation-free days compared to that in the control group. A DP-limited strategy has already been studied in a feasibility trial.^(14)^ A very low tidal volume was used in the DP-limited group to decrease the DP. The main safety concerns were hypercapnia and respiratory acidosis. However, increasing the respiratory rate was sufficient to avoid severe respiratory acidosis in most patients. No safety concerns were raised. The best PEEP titration strategy in patients with ARDS remains controversial.^(11^^,^^15^^,^^16)^ The strategy with the greatest validation in the literature continues to be the PEEP-FiO_2_ table, which was constructed to correlate PEEP values according to FiO_2_ needs.^(8^^,^^9)^ No strategy has been shown to be superior to the PEEP-FiO_2_ table in robust randomized clinical trials.^(17)^ The ART trial was conducted to study prolonged alveolar recruitment with elevated pressures, followed by PEEP titration for best compliance in patients with ARDS. This strategy resulted in increased mortality, mainly due to the occurrence of barotrauma.^(1)^ Nevertheless, the LOVS and EXPRESS studies were performed to test alternative strategies to the PEEP-FiO_2_ table; however, their results did not show superiority.^(18^^,^^19)^ Ventilatory strategies aimed at DP as a target and patient-centered outcomes have not been carried out in well-designed randomized controlled studies to date.^(20^^,^^21)^ The STAMINA trial may show whether this strategy is superior to the current standard of care (PEEP table). If our study shows the benefit of limiting DP by optimizing PEEP guided by compliance of the respiratory system and lowering tidal volume by increasing the number of ventilator-free days, we can contribute to changes in clinical practice and improvements in the treatment of ARDS.

## Supplementary Material



## Roles and responsibilities:

Supplementary Material (Appendix 1)
