# Supplementary material for: Prospective, randomized, controlled trial assessing the effects of a driving pressure–limiting strategy for patients with acute respiratory distress syndrome due to community-acquired pneumonia (STAMINA trial): protocol and statistical analysis plan
Source: Crit Care Sci. 2024 Apr 26;36:e20240210en. doi: 10.62675/2965-2774.20240210-en (PMC11098077; doi:10.62675/2965-2774.20240210-en)
Supplement: Supplementary file 1 [file 2965-2774-ccsci-36-e20240210en-Suppl01.pdf]

Critical Care Science
https://doi.org/10.62675/2965-2774.20240210-en

SUPPLEMENTARY MATERIAL

Prospective, randomized, controlled trial assessing the effects of a driving-pressure limiting strategy for patients with acute respiratory distress syndrome due to community-acquired pneumonia (STAMINA trial): protocol and statistical analysis

STAMINA Study Group Investigators

APPENDIX 1 - ROLES AND RESPONSIBILITIES

**National Coordinating Center:**	Instituto de Pesquisa do Hospital do Coração (IP-Hcor)<br>Rua Desembargador Eliseu Guilherme, 200 - Paraíso<br>ZIP code: 04004-030 - São Paulo (SP), Brazil<br>Tel: 11 3886 4693<br>Fax: 11 3886 4695
**Sponsor:**	*Associação Beneficente Síria* - Hcor
**Vesion:**	6.0. August 01st, 2023
**Trial registration number:**	NCT04972318
**Funding:**	*Programa de Apoio ao Desenvolvimento Institucional do Sistema Único de Saúde* (PROADI-SUS). *Ministério da Saúde* - Brazil

This study results from a collaboration between the Hcor Research Institute and the Brazilian Network for Research in Intensive Medicine (BRICNet).

Roles and Responsabilities

Steering Committee

Fernando Godinho Zampieri, Flavia Ribeiro Machado, Eliana Bernadete Caser, Priscilla Aquino Martins, Marcelo Luiz Pereira Romano, Glauco Adrieno Westphal, Felipe Dal-Pizzol, Viviane Cordeiro Veiga, Cintia Magalhães Carvalho Grion, Rodrigo Santos Biondi, Gustavo Adolfo Ospina-Tascón, Bruno Martins Tomazini, Juliana Carvalho Ferreira, Israel Silva Maia, Alexandre Biasi Cavalcanti.

Responsibilities: study design, protocol development, participant safety and ethics throughout the trial, study implementation oversight, protocol amendments, collaboration and communication between investigators and writing the manuscript.

Trial Sponsor - Hcor Research Institute

Responsibilities: study initiation, protocol development, regulatory compliance, investigator selection and training, trial monitoring and oversight, safety reporting, data management, financial management, quality assurance and quality control, reporting and publication, trial completion and closeout.

This is an open access article under the CC BY license (https://creativecommons.org/licenses/by/4.0/).

Operational Committee - Hcor Research Institute

Roles and responsibilities: study implementation, site selection and management, participant recruitment and retention, data collection and management, training and support, monitoring and quality control, communication and collaboration, adverse event reporting, timelines and milestones, protocol amendments and documentation and trial closeout.

Israel Silva Maia - Principal Investigator<br>E-mail: ismaia@hcor.com.br	
Rodrigo Magalhães Gurgel - Center Management<br>E-mail: rgurgel@hcor.com.br<br>Phone: +55 (11) 3053-6611 - R 8228	Erica Regina Ribeiro Sady - Data Management<br>E-mail: esady@hcor.com.br<br>Phone: +55 (11) 3053-6611 - R 8209
Marina Lazzari Nicola - Center Management<br>E-mail: manicola@hcor.com.br<br>Phone: +55 (11) 3053-6611 - R 8206	Lucas Damiani - Statistician<br>E-mail: ldamiani@hcor.com.br
Letícia Barbante - Regulatory Management<br>E-mail: lbarbante@hcor.com.br<br>Phone: +55 (11) 3053-6611 - R 8236	Júlia Souza de Oliveira - Statistician<br>E-mail: jusoliveira@hcor.com.br<br>Phone: +55 (11) 3053-6611 - R 8236

Department Coordinators - Hcor Research Institute

Ligia Nasi Laranjeira - Manager of the Research Institute<br>E-mail: lnasi@hcor.com.br
Tamiris Abait Miranda - Coordinator of the Center Management Department<br>E-mail: tabait@hcor.com.br
Eliana Santucci - Coordinator of the Data Management Department<br>E-mail: esanticci@hcor.com.br
Nanci Valeis - Manager of the Department of Regulatory Affairs<br>E-mail: nvaleis@hcor.com.br
Lucas Damiani - Coordinator of the Department of Statistics<br>E-mail: ldamiani@hcor.com.br
Karina Leal Negrelli - Coordinator of the Department of Regulatory Affairs<br>E-mail: Knegrelli@hcor.com.br

Data Monitoring Committee

Rinaldo Bellomo, AO MBBS MD FRACP FCICM FAHMS - Professor, Faculty of Medicine, Monash University, Melbourne Australia

Michael Bailey, PhD - Professor of Statistics, Monash University, Melbourne Australia

Paul Young - Professor, Intensive Care specialist, Wellington, New Zealand

APPENDIX 2 - STATISTICAL ANALYSIS PLAN

Prospective, randomized, controlled trial comparing different ventilatory strategies in patients with acute respiratory distress syndrome due to community-acquired pneumonia: STAMINA. Statistical Analysis Plan (SAP)

STAMINA
ventilator STrAtegy for coMmunIty acquired pNeumoniA

**National Coordinating Center:**	Instituto de Pesquisa do Hospital do Coração (IP-Hcor)<br>Rua Desembargador Eliseu Guilherme, 200 - Paraíso<br>ZIP code: 04004-030 - São Paulo (SP), Brazil<br>Tel: 11 3886 4693<br>Fax: 11 3886 4695
**Sponsor:**	*Associação Beneficente Síria* - Hcor
**Protocol version:**	6.0. August 01st, 2023
**SAP version**	1.0. August 01st, 2023
**Trial Registration Number:**	NCT04972318

This study results from a collaboration between the Hcor Research Institute and the Brazilian Network for Research in Intensive Medicine (BRICNet).

Initial Steering Committee:
Fernando Godinho Zampieri, Flávia Ribeiro Machado, Eliana Bernadete Caser, Priscilla Aquino Martins, Marcelo Luiz Pereira Romano, Glauco Adrieno Westphal, Felipe Dal-Pizzol, Viviane Cordeiro Veiga, Cintia Magalhães Carvalho Grion, Rodrigo Santos Biondi, Bruno Martins Tomazini, Juliana Carvalho Ferreira, Israel Silva Maia, Alexandre Biasi Cavalcanti

List of acronyms and abbreviations

IRB	Institutional Review Board
CONEP	National Research Ethics Commission
COVID -19	Coronavirus Disease 2019
DMC	Data Monitoring Committee
DP	driving pressure or distension pressure
AE	Adverse event
SAE	Serious adverse event
FiO_2	Fraction of inspired oxygen
$PaCO_2$	Partial pressure of carbon dioxide
PaO_2	Partial pressure of oxygen
PEEP	Positive end-expiratory pressure
ARDS	Acute respiratory distress syndrome
ICF	Informed Consent Form
ICU	Intensive care unit

INTRODUCTION

The STAMINA study is a randomized, multicenter, open-label trial that compares a driving pressure-limiting strategy to the ARDSNet low-positive end-expiratory pressure (PEEP) table in patients with moderate-to-severe acute respiratory distress syndrome (ARDS) secondary to community acquired pneumonia, who are hospitalized in intensive care units (ICU) and are under mechanical ventilatory support. It is expected to include 500 patients in at least 20 Brazilian ICUs and 1 Colombian ICU for at least 36 months. Eligible patients will receive the standard ventilatory strategy (ARDSNet) *versus* a compliance-guided PEEP optimization strategy associated with reduced driving pressure (DP).

Details about the intervention and comparator group are found in the study protocol. This document describes the statistical analysis plan for the study.

Objectives

Primary objective

To evaluate whether a driving pressure limiting strategy titrating PEEP according to best compliance is superior in terms of increasing the ventilator-free days to the standard strategy using low PEEP table.

Secondary objectives

To evaluate whether a driving pressure limiting strategy titrating PEEP according to best compliance is superior to the standard strategy using PEEP table and inspired oxygen fraction in relation to ventilatory mechanics and oxygenation parameters.

Eligibility criteria

Inclusion criteria

1. Patients with pneumonia on invasive mechanical ventilation (patients where the indication of intubation was respiratory failure secondary to pneumonia);
2. Acute bilateral infiltrate of non-exclusively cardiogenic origin, at the judgment of the attending physician;
3. One of the below:
   a. Inspired oxygen fraction above 50% with PEEP of at least 8cmH_2O to maintain saturation above 93% **OR**
   b. PaO_2/FiO_2 ratio < 200 with PEEP > 5cmH_2O.

Exclusion criteria

1. Patients with inclusion criteria for more than 36 hours;
2. Intracranial hypertension or acute neurological disease (stroke, subarachnoid hemorrhage;
3. Refusal of the legal representative;
4. Patients under 18 years of age;
5. Patients considered not candidates for measures of full invasive support at the time of randomization, that is, patients who have some clear definition, at the time of randomization, of not instituting other invasive supports;
6. Patient with aerial fistula or barotrauma;
7. Patients with a history of home use of oxygen by chronic respiratory disease.

Note: The inclusion criteria 3a assumes the need for these parameters to maintain at least 93% oxygen saturation. If the patient is admitted with a high inspired oxygen fraction and/or high PEEP, it is recommended, if possible, to try to reduce ventilatory parameters (FiO2 and/or PEEP) to assess the SpO_2 response, before considering this criterion fulfilled.

Outcomes

Primary

Ventilator-free days within 28 days from randomization or until hospital discharge measured as follows:

- D = zero (if the patient dies within 28 days in the hospital or remains on respiratory support with mechanical ventilation ≥ 28 days)
- D = 28 - x (if the patient is released from the hospital in < 28 days, where x represents the number of days with mechanical ventilation during hospitalization)

The number of days on mechanical ventilation will be counted as every day the patient spent at least 12 hours on mechanical ventilation. If there is an interruption of mechanical ventilation followed by restart within 48 hours (extubation failure), the entire period will be computed as a single period. For tracheostomized patients, the same criterion is valid. One day of ventilation is computed whenever the patient persists more than 12 hours ventilated. The number of days on mechanical ventilation will be counted as every day the patient spent at least 12 hours on mechanical ventilation. If there is an interruption of mechanical ventilation followed by restart within 48 hours (extubation failure), the entire period will be computed as a single period.

For tracheostomized patients, the same criterion is valid. Short periods of nebulization will be accounted for, and one day of ventilation is computed whenever the patient persists more than 12 hours ventilated.

Secondary

1. In hospital mortality within 90 days;
2. ICU mortality within 90 days;
3. Need for rescue therapies within 28 days (extracorporeal circulation, recruitment maneuver, inhaled nitric oxide).

Exploratory

1. Oxygenation, measured by the PaO_2/FiO_2 ratio during the first 3 days;
2. Value of driving pressure (DP) during the first 3 days;
3. Ventilatory ratio, defined as $PaCO_2$ multiplied by minute ventilation divided by 100 × ideal weight in kilograms × 37.5, measured in the first 3 days;
4. Oxygenation index, defined by mean airway pressure (MAP) × FiO_2 × 100 ÷ PaO_2, measured in the first 3 days;
5. Mechanical power, defined by energy transfer (0.098) x respiratory rate x tidal volume x [peak airway pressure – 0.5 x (plateau pressure – PEEP)], measured in the first 3 days;
6. ICU-free days in 28 days;
7. ICU length of stay;
8. Hospital length of stay.

Safety outcomes

1. Occurrence of barotrauma (subcutaneous emphysema, pneumothorax, pneumatocele or pneumomediastinum);
2. Other serious adverse events possibly related to mechanical ventilation.

Randomization

The randomization list will be generated electronically using appropriate software. Randomization will be performed in blocks (variable blocks) stratified by center and diagnosis of COVID-19. The confidentiality of the randomization list will be maintained through the automated randomization system, central, via internet, available 24 hours a day (RedCap). The group in which the patient will be allocated will only be disclosed after registering the information in the electronic system, which prevents the investigator and the assistant team from predicting which treatment groups the patient will be allocated. The investigator must visit the website used in the study (RedCap) to formally allocate the patient to the different treatment groups.

Blinding

There will be no blinding for patients, clinicians and outcome assessors, however, the outcome assessors will have access to outcomes data only after the end of the study.

Statistical analysis

All analyses will adhere to the principle of a modified intention-to-treat approach. Exclusions of participants after randomization may occur due to refusals, given the retrospective (opt-out) nature of the informed consent. They will be carried out with using R software in a version equal to or greater than 4.0.1 (R Core Team, 2021, Viena, Áustria[1]).

Sample size

The trial will randomize 500 patients in a 1:1 allocation (250 in each group). This number of patients provides a power of at least 90% for difference around 3-days on mechanical ventilation free days and a 5% lower mortality rate in the treatment group.

Considering the raw data from the non-death-related part of the composite outcome (ventilator-free days) and a mortality rate of 60% in the Control group of the CoDEX study[2] as a reference, our simulations reached an average of 4.7 mechanical ventilation free days in 28 days for the Control group, with quartiles [3.7 - 5.8], standard deviation of 8.2; and mean of 7.8 mechanical ventilation free days for the Treatment group, with quartiles [6.5 - 9.2] and standard deviation 10.5.

Estimates of the study power were performed from 2,000 simulations of different scenarios for the primary outcome, considering that death within 28 days would imply zero ventilator-free days, even if the patient was

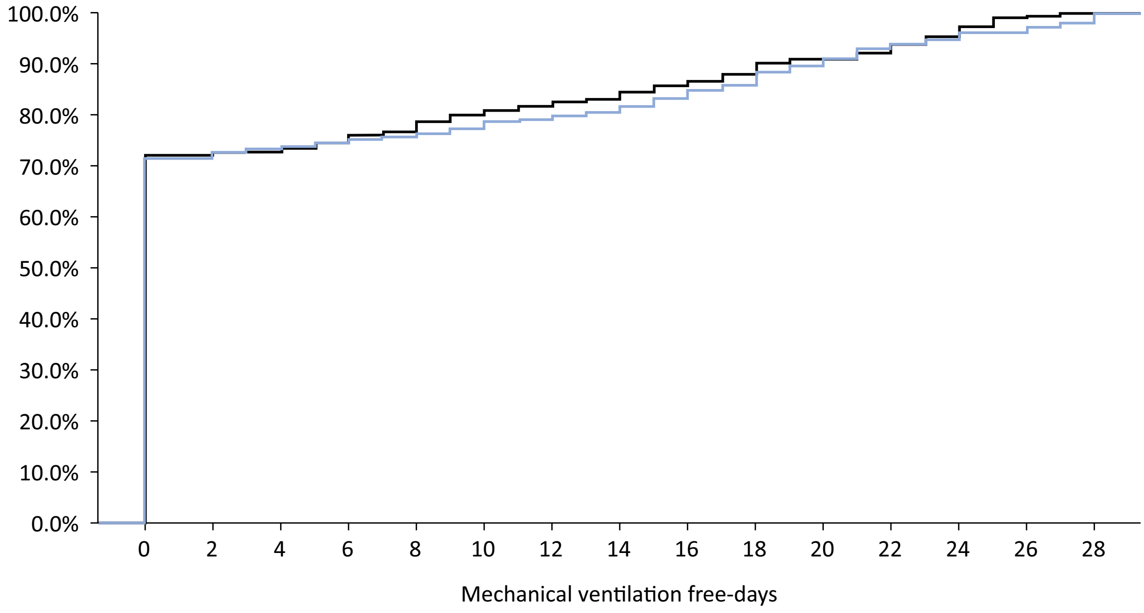

Figure 1 - Cumulative distribution for mechanical ventilator-free days up to 28 days. Null scenario.

ventilator-free for at least one day during hospitalization. Both groups were compared using proportional odds logistic regression model (ordinal outcome).

Figure 1 shows the cumulative distribution for the primary outcome from a simulation considering the null model (effect of the Treatment group identical to the Control group) to better describe the expected distribution of the primary outcome.

Interim analysis

A Data and Safety Monitoring Committee (DMC) will be formed by epidemiologists and intensivists independent from study investigators. The DMC is responsible for providing guidance to the Steering Committee on whether to continue the study as planned or discontinue recruitment based on evidence that the experimental group intervention results in increased mortality compared to the control. At the beginning of its activities, the DMC must prepare a charter specifying the details of its formation, its functioning, meetings and interruption rules. In any case, the rules of the booklet should be guided by the principles described below.

Two interim analysis are planned:

1. Once 100 patients complete 28 days of follow-up, preliminary safety data will be shared with the DMC, along with treatment adherence results (Control/ARDSNET and Intervention). In this interim analysis, efficacy outcomes will not be evaluated, and therefore, the study cannot be interrupted for benefit.
2. Once 200 patients complete 28 days of follow-up, the DMC will assess the results of all outcomes in a non-blind manner (safety and efficacy). In light of the review of adverse events and external evidence, the Data Monitoring Committee should assess whether there is evidence beyond reasonable doubt that one of the interventions is clearly contraindicated for all patients or some subgroup. For interruptions for efficacy (superiority) or safety based on the ventilator-free days (primary outcome), it is suggested that the DMC consider the Haybittle-Peto criterion (p value <0.001).[3]

As the Haybittle-Peto criterion[3] is rigorous, there is practically no consumption of type I error, and thus the final evaluation is maintained considering a significance level fixed at 5%.

Baseline data presentation

Baseline characteristics of available patients by group will be described as shown in table 1S.

Adherence data presentation

The interventions follow different criteria for adjusting the mechanical ventilators' parameters and will be presented independently as described in tables 2S (Control/ARDSNET) and 3S (Intervention/STAMINA). Table 4S describes the results of ventilatory parameters, blood pressure, sedation, rescue therapy, laboratory parameters in the first 72 hours of intervention.

Table 1S - Baseline characteristics of study participants

	Intervention - STAMINA (xx)	Control – ARDSNet (n=xx)
Age, Median [Quartiles] (no.)	xx [xx - xx] (n = xx)	xx [xx - xx] (n = xx)
Women (sex at birth), no./N (%)	xx/xx (xx)	xx/xx (xx)
Comorbidities, no./N (%)		
Diabetes	xx/xx (xx)	xx/xx (xx)
Chronic obstructive pulmonary disease	xx/xx (xx)	xx/xx (xx)
Heart failure	xx/xx (xx)	xx/xx (xx)
Percutaneous coronary intervention with stent or chest pain	xx/xx (xx)	xx/xx (xx)
Acute myocardial infarction	xx/xx (xx)	xx/xx (xx)
Arterial hypertension	xx/xx (xx)	xx/xx (xx)
Peripheral arterial disease	xx/xx (xx)	xx/xx (xx)
Transient ischemic attack or stroke	xx/xx (xx)	xx/xx (xx)
Stroke with neurologic deficit	xx/xx (xx)	xx/xx (xx)
Sensorial deficit	xx/xx (xx)	xx/xx (xx)
Status - nonindependent functional status	xx/xx (xx)	xx/xx (xx)
Hematological malignancies or solid tumor	xx/xx (xx)	xx/xx (xx)
Chronic Kidney Disease	xx/xx (xx)	xx/xx (xx)
Modified frailty index score, Median [Quartiles]	xx [xx - xx] (n = xx)	xx [xx - xx] (n = xx)
Status - COVID-19, no./N (%)		
Pneumonia COVID-19	xx/xx (xx)	xx/xx (xx)
Pneumonia non COVID-19	xx/xx (xx)	xx/xx (xx)
Ventilatory support, Median [Quartiles]		
Days of mechanical ventilation prior to randomization	xx [xx - xx] (n = xx)	xx [xx - xx] (n = xx)
Tidal volume/predicted body weight (mL/Kg)	xx [xx - xx] (n = xx)	xx [xx - xx] (n = xx)
Positive end-expiratory pressure (cmH_2O)	xx [xx - xx] (n = xx)	xx [xx - xx] (n = xx)
FiO_2 (%)	xx [xx - xx] (n = xx)	xx [xx - xx] (n = xx)
Respiratory rate (breaths/minute)	xx [xx - xx] (n = xx)	xx [xx - xx] (n = xx)
Plateau airway pressure (cmH_2O)	xx [xx - xx] (n = xx)	xx [xx - xx] (n = xx)
Respiratory system static compliance (mL/cmH_2O)	xx [xx - xx] (n = xx)	xx [xx - xx] (n = xx)
Driving pressure (cmH_2O)	xx [xx - xx] (n = xx)	xx [xx - xx] (n = xx)
Minute ventilation (L/minute)	xx [xx - xx] (n = xx)	xx [xx - xx] (n = xx)
Arterial blood gases, Median [Quartiles] (no.)		
pH	x.xx [x.xx - x.xx] (n = xx)	x.xx [x.xx - x.xx] (n = xx)
PaO_2 (mmHg)	xx [xx - xx] (n = xx)	xx [xx - xx] (n = xx)
$PaCO_2$ (mmHg)	xx [xx - xx] (n = xx)	xx [xx - xx] (n = xx)
Bicarbonate (mmol/L)	xx [xx - xx] (n = xx)	xx [xx - xx] (n = xx)
Base Excess	xx [xx - xx] (n = xx)	xx [xx - xx] (n = xx)
SaO_2 (%)	xx [xx - xx] (n = xx)	xx [xx - xx] (n = xx)
$PaO_2:FiO_2$	xx [xx - xx] (n = xx)	xx [xx - xx] (n = xx)
Prone position, no./N (%)	xx/xx (xx)	xx/xx (xx)
Vasoactive drugs use, no./N (%)		
Norepinephrine	xx/xx (xx)	xx/xx (xx)
Epinephrine	xx/xx (xx)	xx/xx (xx)
Vasopressin	xx/xx (xx)	xx/xx (xx)
Laboratory tests, Median [Quartiles] (no.)		
Creatinine (mg/dL)	x.x [x.x - x.x] (n = xx)	x.x [x.x - x.x] (n = xx)
Total bilirubin (mg/dL)	x.x [x.x - x.x] (n = xx)	x.x [x.x - x.x] (n = xx)
Platelets (mil/mm^3)	x.x [x.x - x.x] (n = xx)	x.x [x.x - x.x] (n = xx)

FiO_2 - fraction of inspired oxygen; PaO_2 - partial pressure of arterial oxygen; $PaCO_2$ - partial pressure of arterial carbon dioxide; SaO_2 - arterial oxy-hemoglobin saturation.

Table 2S - ARDSNet treatment adherence indicators (Control)

	PEEP titration using the PEEP table strategy (Low PEEP) – ARDSNET Group (n = 250)			
	Day 0	Day 1	Day 2	Day 3
No. of patients	xx	xx	xx	xx
Neuromuscular blockade pre titration of PEEP by the PEEP/FiO2 strategy table (Low PEEP), No. (%)	xx (xx.x)	xx (xx.x)	xx (xx.x)	xx (xx.x)
Deep sedation (RASS -4 or -5) before PEEP titration, No./Total No. (%)	xx (xx.x)	xx (xx.x)	xx (xx.x)	xx (xx.x)
Titrated PEEP (cmH_2O) mean (SD)	xx (xx.x)	xx (xx.x)	xx (xx.x)	xx (xx.x)
Titrated FiO_2 (%) mean (SD)	xx (xx.x)	xx (xx.x)	xx (xx.x)	xx (xx.x)
Compliance to protective ventilation maintenance on 8 hours post PEEP titration maneuver				
Tidal volume/predicted body weight (mL/kg)				
Median [Quartiles] (no.)	x.xx [x.xx - x.xx] (n = xx)	x.xx [x.xx - x.xx] (n = xx)	x.xx [x.xx - x.xx] (n = xx)	x.xx [x.xx - x.xx] (n = xx)
No. of participants who adhered to the parameter ≤ 6mL/kg /Total (%)	xx (xx)	xx (xx)	xx (xx)	xx (xx)
Plateau pressure				
Median [Quartiles] (no.)	x.xx [x.xx - x.xx] (n = xx)	x.xx [x.xx - x.xx] (n = xx)	x.xx [x.xx - x.xx] (n = xx)	x.xx [x.xx - x.xx] (n = xx)
No. of participants who adhered to the parameter ≤ 30cmH_2O /Total (%)	xx (xx)	xx (xx)	xx (xx)	xx (xx)
Compliance to protective ventilation maintenance on 16 hours post PEEP titration maneuver				
Tidal volume/predicted body weight (mL/Kg)				
Median [Quartiles] (no.)	x.xx [x.xx - x.xx] (n = xx)	x.xx [x.xx - x.xx] (n = xx)	x.xx [x.xx - x.xx] (n = xx)	x.xx [x.xx - x.xx] (n = xx)
No. of participants who adhered to the parameter ≤ 6mL/Kg /Total (%)	xx (xx)	xx (xx)	xx (xx)	xx (xx)
Plateau pressure				
Median [Quartiles] (no.)	x.xx [x.xx - x.xx] (n = xx)	x.xx [x.xx - x.xx] (n = xx)	x.xx [x.xx - x.xx] (n = xx)	x.xx [x.xx - x.xx] (n = xx)
No. of participants who adhered to the parameter ≤ 30cmH_2O /Total (%)	xx (xx)	xx (xx)	xx (xx)	xx (xx)
Compliance to protective ventilation maintenance on 24 hours post PEEP titration maneuver				
Tidal volume/ predicted body weight (mL/kg)				
Median [Quartiles] (no.)	x.xx [x.xx - x.xx] (n = xx)	x.xx [x.xx - x.xx] (n = xx)	x.xx [x.xx - x.xx] (n = xx)	x.xx [x.xx - x.xx] (n = xx)
No. of participants who adhered to the parameter ≤ 6mL/kg /Total (%)	xx (xx)	xx (xx)	xx (xx)	xx (xx)
Plateau pressure				
Median [Quartiles] (no.)	x.xx [x.xx - x.xx] (n = xx)	x.xx [x.xx - x.xx] (n = xx)	x.xx [x.xx - x.xx] (n = xx)	x.xx [x.xx - x.xx] (n = xx)
No. of participants who adhered to the parameter ≤ 30cmH_2O/Total (%)	xx (xx)	xx (xx)	xx (xx)	xx (xx)
PEEP titration manuever repeated a day, No./No. total (%)				
Once	xx/xx (xx.x)	xx/xx (xx.x)	xx/xx (xx.x)	xx/xx (xx.x)
Twice	xx/xx (xx.x)	xx/xx (xx.x)	xx/xx (xx.x)	xx/xx (xx.x)
Thrice	xx/xx (xx.x)	xx/xx (xx.x)	xx/xx (xx.x)	xx/xx (xx.x)
Spontaneous breathing test, No./Total No. (%)	xx/xx (xx.x)	xx/xx (xx.x)	xx/xx (xx.x)	xx/xx (xx.x)

PEEP - positive end-expiratory pressure; FiO_2 - fraction of inspired oxygen; RASS - Richmond Agitation-Sedation Scale.

Table 3S - STAMINA treatment adherence indicators (Intervention)

	PEEP titration maneuver - STAMINA Group (n = 250)			
	Day 0	Day 1	Day 2	Day 3
Neuromuscular blockade before PEEP titration, No./Total No. (%)	xx (xx.x)	xx (xx.x)	xx (xx.x)	xx (xx.x)
Deep sedation (RASS -4 or -5) before PEEP titration, No./Total No. (%)	xx (xx.x)	xx (xx.x)	xx (xx.x)	xx (xx.x)
Maximum PEEP in the incremental step of the PEEP titration maneuver, No. /Total No (%)				
Maximum (PEEP = 20cmH_2O)	xx (xx.x)	xx (xx.x)	xx (xx.x)	xx (xx.x)
Interrupted at PEEP = 18cmH_2O	xx (xx.x)	xx (xx.x)	xx (xx.x)	xx (xx.x)
Interrupted at PEEP = 16cmH_2O	xx (xx.x)	xx (xx.x)	xx (xx.x)	xx (xx.x)
Interrupted at PEEP = 14cmH_2O	xx (xx.x)	xx (xx.x)	xx (xx.x)	xx (xx.x)
Interrupted at PEEP = 12cmH_2O	xx (xx.x)	xx (xx.x)	xx (xx.x)	xx (xx.x)
Interrupted at PEEP = 10cmH_2O	xx (xx.x)	xx (xx.x)	xx (xx.x)	xx (xx.x)
Intervention not performed	xx (xx.x)	xx (xx.x)	xx (xx.x)	xx (xx.x)
Titrated PEEP (cmH_2O), mean (SD)	xx (xx.x)	xx (xx.x)	xx (xx.x)	xx (xx.x)
Reason for interruption of the incremental step of the PEEP titration maneuver, No. of events (%)				
Peak Pressure ≥ 40cmH2O (sustained ≥ 4 cycles)	xx (xx.x)	xx (xx.x)	xx (xx.x)	xx (xx.x)
Plateau Pressure ≥ 33cmH_2O (sustained ≥ 4 cycles)	xx (xx.x)	xx (xx.x)	xx (xx.x)	xx (xx.x)
Unstable cardiac arrhythmia	xx (xx.x)	xx (xx.x)	xx (xx.x)	xx (xx.x)
Severe arterial hypotension, requiring an increase in the vasopressor dose	xx (xx.x)	xx (xx.x)	xx (xx.x)	xx (xx.x)
Compliance to protective ventilation maintenance on 8 hours post PEEP titration maneuver				
Tidal volume/Predicted weight				
Median [Quartiles] (no.)	x.xx [x.xx - x.xx] (n = xx)	x.xx [x.xx - x.xx] (n = xx)	x.xx [x.xx - x.xx] (n = xx)	x.xx [x.xx - x.xx] (n = xx)
No. of participants who adhered to the parameter ≤ 6mL/kg /Total (%)	xx (xx)	xx (xx)	xx (xx)	xx (xx)
Plateau pressure				
Median [Quartiles] (no.)	x.xx [x.xx - x.xx] (n = xx)	x.xx [x.xx - x.xx] (n = xx)	x.xx [x.xx - x.xx] (n = xx)	x.xx [x.xx - x.xx] (n = xx)
No. of participants who adhered to the parameter ≤ 30cmH_2O /Total (%)	xx (xx)	xx (xx)	xx (xx)	xx (xx)
Driving pressure				
Median [Quartiles] (no.)	x.xx [x.xx - x.xx] (n = xx)	x.xx [x.xx - x.xx] (n = xx)	x.xx [x.xx - x.xx] (n = xx)	x.xx [x.xx - x.xx] (n = xx)
No. of participants who adhered to the parameter ≤ 14cmH_2O /Total (%)	xx (xx)	xx (xx)	xx (xx)	xx (xx)
Compliance to protective ventilation maintenance on 16 hours post PEEP titration maneuver				
Tidal volume/Predicted weight				
Median [Quartiles] (no.)	x.xx [x.xx - x.xx] (n = xx)	x.xx [x.xx - x.xx] (n = xx)	x.xx [x.xx - x.xx] (n = xx)	x.xx [x.xx - x.xx] (n = xx)
No. of participants who adhered to the parameter ≤ 6mL/kg /Total (%)	xx (xx)	xx (xx)	xx (xx)	xx (xx)
Plateau pressure				
Median [Quartiles] (no.)	x.xx [x.xx - x.xx] (n = xx)	x.xx [x.xx - x.xx] (n = xx)	x.xx [x.xx - x.xx] (n = xx)	x.xx [x.xx - x.xx] (n = xx)
No. of participants who adhered to the parameter ≤ 30cmH_2O /Total (%)	xx (xx)	xx (xx)	xx (xx)	xx (xx)
Driving pressure				
Median [Quartiles] (no.)	x.xx [x.xx - x.xx] (n = xx)	x.xx [x.xx - x.xx] (n = xx)	x.xx [x.xx - x.xx] (n = xx)	x.xx [x.xx - x.xx] (n = xx)

Continue...

...continuation

	PEEP titration maneuver - STAMINA Group (n = 250)			
	Day 0	Day 1	Day 2	Day 3
No. of participants who adhered to the parameter ≤ 14cmH_2O /Total (%)	xx (xx)	xx (xx)	xx (xx)	xx (xx)
Compliance to protective ventilation maintenance on 24 hours post PEEP titration maneuver				
Tidal volume/Predicted weight				
Median [Quartiles] (no.)	x.xx [x.xx - x.xx] (n = xx)	x.xx [x.xx - x.xx] (n = xx)	x.xx [x.xx - x.xx] (n = xx)	x.xx [x.xx - x.xx] (n = xx)
No. of participants who adhered to the parameter ≤ 6mL/kg /Total (%)	xx (xx)	xx (xx)	xx (xx)	xx (xx)
Plateau pressure				
Median [Quartiles] (no.)	x.xx [x.xx - x.xx] (n = xx)	x.xx [x.xx - x.xx] (n = xx)	x.xx [x.xx - x.xx] (n = xx)	x.xx [x.xx - x.xx] (n = xx)
No. of participants who adhered to the parameter ≤ 30cmH_2O /Total (%)	xx (xx)	xx (xx)	xx (xx)	xx (xx)
Driving pressure				
Median [Quartiles] (no.)	x.xx [x.xx - x.xx] (n = xx)	x.xx [x.xx - x.xx] (n = xx)	x.xx [x.xx - x.xx] (n = xx)	x.xx [x.xx - x.xx] (n = xx)
No. of participants who adhered to the parameter ≤ 14 cmH_2O /Total (%)	xx (xx)	xx (xx)	xx (xx)	xx (xx)
PEEP titration manuever repeated a day, No./No. total (%)				
Once	xx/xx (xx.x)	xx/xx (xx.x)	xx/xx (xx.x)	xx/xx (xx.x)
Twice	xx/xx (xx.x)	xx/xx (xx.x)	xx/xx (xx.x)	xx/xx (xx.x)
Thrice	xx/xx (xx.x)	xx/xx (xx.x)	xx/xx (xx.x)	xx/xx (xx.x)
Spontaneous breathing test, No./Total No. (%)	xx/xx (xx.x)	xx/xx (xx.x)	xx/xx (xx.x)	xx/xx (xx.x)

PEEP - positive end-expiratory pressure; RASS - Richmond Agitation-Sedation Scale.

Table 4S - Daily ventilatory parameters, arterial blood gases, level of sedation, use of prone position and laboratory parameter from Day 0 through Day 3

	Day 0 - Baseline		Day 0*		Day 1*		Day 2*		Day 3*	
	STAMINA	ARDSNet	STAMINA	ARDSNet	STAMINA	ARDSNet	STAMINA	ARDSNet	STAMINA	ARDSNet
No. of patients	xx	xx	xx	xx	xx	xx	xx	xx	xx	xx
Mechanical ventilation parameters										
Before intervention										
Tidal volume/Predicted weight ≤ 6mL/Kg, mean (SD)	xx (xx.x)	xx (xx.x)	xx (xx.x)	xx (xx.x)	xx (xx.x)	xx (xx.x)	xx (xx.x)	xx (xx.x)	xx (xx.x)	xx (xx.x)
PEEP (cmH_2O), mean (SD)	xx (xx.x)	xx (xx.x)	xx (xx.x)	xx (xx.x)	xx (xx.x)	xx (xx.x)	xx (xx.x)	xx (xx.x)	xx (xx.x)	xx (xx.x)
FiO_2 (%), mean (SD))	xx (xx.x)	xx (xx.x)	xx (xx.x)	xx (xx.x)	xx (xx.x)	xx (xx.x)	xx (xx.x)	xx (xx.x)	xx (xx.x)	xx (xx.x)
Respiratory rate (breaths/min), mean (SD)	xx (xx.x)	xx (xx.x)	xx (xx.x)	xx (xx.x)	xx (xx.x)	xx (xx.x)	xx (xx.x)	xx (xx.x)	xx (xx.x)	xx (xx.x)
Minute ventilation (L/min), mean (SD)	xx (xx.x)	xx (xx.x)	xx (xx.x)	xx (xx.x)	xx (xx.x)	xx (xx.x)	xx (xx.x)	xx (xx.x)	xx (xx.x)	xx (xx.x)
Plateau Pressure (cmH_2O), mean (SD)	xx (xx.x)	xx (xx.x)	xx (xx.x)	xx (xx.x)	xx (xx.x)	xx (xx.x)	xx (xx.x)	xx (xx.x)	xx (xx.x)	xx (xx.x)
Static compliance of the respiratory system (mL/cmH_2O), mean (SD)	xx (xx.x)	xx (xx.x)	xx (xx.x)	xx (xx.x)	xx (xx.x)	xx (xx.x)	xx (xx.x)	xx (xx.x)	xx (xx.x)	xx (xx.x)
Driving pressure (cmH_2O), mean (SD)	xx (xx.x)	xx (xx.x)	xx (xx.x)	xx (xx.x)	xx (xx.x)	xx (xx.x)	xx (xx.x)	xx (xx.x)	xx (xx.x)	xx (xx.x)
After intervention										
Tidal Volume/Predicted Weight ≤ 6mL/kg, mean (SD)	NaN ± NA (n = 0)	NaN ± NA (n = 0)	xx (xx.x)	xx (xx.x)	xx (xx.x)	xx (xx.x)	xx (xx.x)	xx (xx.x)	xx (xx.x)	xx (xx.x)
PEEP (cmH_2O), mean (SD)	NaN ± NA (n = 0)	NaN ± NA (n = 0)	xx (xx.x)	xx (xx.x)	xx (xx.x)	xx (xx.x)	xx (xx.x)	xx (xx.x)	xx (xx.x)	xx (xx.x)
FiO_2 (%), mean (SD))	NaN ± NA (n = 0)	NaN ± NA (n = 0)	xx (xx.x)	xx (xx.x)	xx (xx.x)	xx (xx.x)	xx (xx.x)	xx (xx.x)	xx (xx.x)	xx (xx.x)
Respiratory rate (breaths/minute), mean (SD)	NaN ± NA (n = 0)	NaN ± NA (n = 0)	xx (xx.x)	xx (xx.x)	xx (xx.x)	xx (xx.x)	xx (xx.x)	xx (xx.x)	xx (xx.x)	xx (xx.x)
Minute ventilation (L/minute), mean (SD)	NaN ± NA (n = 0)	NaN ± NA (n = 0)	xx (xx.x)	xx (xx.x)	xx (xx.x)	xx (xx.x)	xx (xx.x)	xx (xx.x)	xx (xx.x)	xx (xx.x)
Plateau Pressure (cmH_2O), mean (SD)	NaN ± NA (n = 0)	NaN ± NA (n = 0)	xx (xx.x)	xx (xx.x)	xx (xx.x)	xx (xx.x)	xx (xx.x)	xx (xx.x)	xx (xx.x)	xx (xx.x)
Static compliance of the respiratory system (mL/cmH_2O), mean (SD)	NaN ± NA (n = 0)	NaN ± NA (n = 0)	xx (xx.x)	xx (xx.x)	xx (xx.x)	xx (xx.x)	xx (xx.x)	xx (xx.x)	xx (xx.x)	xx (xx.x)
Driving pressure (cmH_2O), mean (SD)	NaN ± NA (n = 0)	NaN ± NA (n = 0)	xx (xx.x)	xx (xx.x)	xx (xx.x)	xx (xx.x)	xx (xx.x)	xx (xx.x)	xx (xx.x)	xx (xx.x)
Arterial blood gas analysis, mean (SD)										
Before intervention										
pH	xx (xx.x)	xx (xx.x)	xx (xx.x)	xx (xx.x)	xx (xx.x)	xx (xx.x)	xx (xx.x)	xx (xx.x)	xx (xx.x)	xx (xx.x)
PaO_2 (mmHg)	xx (xx.x)	xx (xx.x)	xx (xx.x)	xx (xx.x)	xx (xx.x)	xx (xx.x)	xx (xx.x)	xx (xx.x)	xx (xx.x)	xx (xx.x)
$PaCO_2$ (mmHg)	xx (xx.x)	xx (xx.x)	xx (xx.x)	xx (xx.x)	xx (xx.x)	xx (xx.x)	xx (xx.x)	xx (xx.x)	xx (xx.x)	xx (xx.x)
Bicarbonate (mmol/L)	xx (xx.x)	xx (xx.x)	xx (xx.x)	xx (xx.x)	xx (xx.x)	xx (xx.x)	xx (xx.x)	xx (xx.x)	xx (xx.x)	xx (xx.x)
Base excess	xx (xx.x)	xx (xx.x)	xx (xx.x)	xx (xx.x)	xx (xx.x)	xx (xx.x)	xx (xx.x)	xx (xx.x)	xx (xx.x)	xx (xx.x)
SaO_2 (%)	xx (xx.x)	xx (xx.x)	xx (xx.x)	xx (xx.x)	xx (xx.x)	xx (xx.x)	xx (xx.x)	xx (xx.x)	xx (xx.x)	xx (xx.x)
PaO_2/FiO_2	xx (xx.x)	xx (xx.x)	xx (xx.x)	xx (xx.x)	xx (xx.x)	xx (xx.x)	xx (xx.x)	xx (xx.x)	xx (xx.x)	xx (xx.x)
Post intervention										
pH	NaN ± NA (n = 0)	NaN ± NA (n = 0)	xx (xx.x)	xx (xx.x)	xx (xx.x)	xx (xx.x)	xx (xx.x)	xx (xx.x)	xx (xx.x)	xx (xx.x)
PaO_2 (mmHg)	NaN ± NA (n = 0)	NaN ± NA (n = 0)	xx (xx.x)	xx (xx.x)	xx (xx.x)	xx (xx.x)	xx (xx.x)	xx (xx.x)	xx (xx.x)	xx (xx.x)

Continue...

...continuation

	Day 0 - Baseline		Day 0*		Day 1*		Day 2*		Day 3*	
	STAMINA	ARDSNet	STAMINA	ARDSNet	STAMINA	ARDSNet	STAMINA	ARDSNet	STAMINA	ARDSNet
$PaCO_2$ (mmHg)	NaN ± NA (n = 0)	NaN ± NA (n = 0)	xx (xx.x)	xx (xx.x)	xx (xx.x)	xx (xx.x)	xx (xx.x)	xx (xx.x)	xx (xx.x)	xx (xx.x)
Bicarbonate (mmol/L)	NaN ± NA (n = 0)	NaN ± NA (n = 0)	xx (xx.x)	xx (xx.x)	xx (xx.x)	xx (xx.x)	xx (xx.x)	xx (xx.x)	xx (xx.x)	xx (xx.x)
Base excess	NaN ± NA (n = 0)	NaN ± NA (n = 0)	xx (xx.x)	xx (xx.x)	xx (xx.x)	xx (xx.x)	xx (xx.x)	xx (xx.x)	xx (xx.x)	xx (xx.x)
SaO_2 (%)	NaN ± NA (n = 0)	NaN ± NA (n = 0)	xx (xx.x)	xx (xx.x)	xx (xx.x)	xx (xx.x)	xx (xx.x)	xx (xx.x)	xx (xx.x)	xx (xx.x)
PaO_2/FiO_2	NaN ± NA (n = 0)	NaN ± NA (n = 0)	xx (xx.x)	xx (xx.x)	xx (xx.x)	xx (xx.x)	xx (xx.x)	xx (xx.x)	xx (xx.x)	xx (xx.x)
Lowest level of sedation (RASS) per day (D0 - D3), No./Total No. (%)										
Alert (RASS ≥ 0)	xx/xx (xx.x)	xx/xx (xx.x)	xx/xx (xx.x)	xx/xx (xx.x)	xx/xx (xx.x)	xx/xx (xx.x)	xx/xx (xx.x)	xx/xx (xx.x)	xx/xx (xx.x)	xx/xx (xx.x)
Lethargic (RASS -1)	xx/xx (xx.x)	xx/xx (xx.x)	xx/xx (xx.x)	xx/xx (xx.x)	xx/xx (xx.x)	xx/xx (xx.x)	xx/xx (xx.x)	xx/xx (xx.x)	xx/xx (xx.x)	xx/xx (xx.x)
Light sedation (RASS -2)	xx/xx (xx.x)	xx/xx (xx.x)	xx/xx (xx.x)	xx/xx (xx.x)	xx/xx (xx.x)	xx/xx (xx.x)	xx/xx (xx.x)	xx/xx (xx.x)	xx/xx (xx.x)	xx/xx (xx.x)
Moderate sedation (RASS -3)	xx/xx (xx.x)	xx/xx (xx.x)	xx/xx (xx.x)	xx/xx (xx.x)	xx/xx (xx.x)	xx/xx (xx.x)	xx/xx (xx.x)	xx/xx (xx.x)	xx/xx (xx.x)	xx/xx (xx.x)
Deep sedation (RASS -4 or -5)	xx/xx (xx.x)	xx/xx (xx.x)	xx/xx (xx.x)	xx/xx (xx.x)	xx/xx (xx.x)	xx/xx (xx.x)	xx/xx (xx.x)	xx/xx (xx.x)	xx/xx (xx.x)	xx/xx (xx.x)
Prone position, No./No. total (%)	xx/xx (xx.x)	xx/xx (xx.x)	xx/xx (xx.x)	xx/xx (xx.x)	xx/xx (xx.x)	xx/xx (xx.x)	xx/xx (xx.x)	xx/xx (xx.x)	xx/xx (xx.x)	xx/xx (xx.x)
Vasopressor use, no./N (%)										
Norepinephrine	xx (xx.x)	xx (xx.x)	xx (xx.x)	xx (xx.x)	xx (xx.x)	xx (xx.x)	xx (xx.x)	xx (xx.x)	xx (xx.x)	xx (xx.x)
Epinephrine	xx (xx.x)	xx (xx.x)	xx (xx.x)	xx (xx.x)	xx (xx.x)	xx (xx.x)	xx (xx.x)	xx (xx.x)	xx (xx.x)	xx (xx.x)
Vasopressin	xx (xx.x)	xx (xx.x)	xx (xx.x)	xx (xx.x)	xx (xx.x)	xx (xx.x)	xx (xx.x)	xx (xx.x)	xx (xx.x)	xx (xx.x)
Laboratory parameters, mean (SD)										
Creatinine (mg/dL)	xx (xx.x)	xx (xx.x)	xx (xx.x)	xx (xx.x)	xx (xx.x)	xx (xx.x)	xx (xx.x)	xx (xx.x)	xx (xx.x)	xx (xx.x)
Bilirubin (mg/dL)	xx (xx.x)	xx (xx.x)	xx (xx.x)	xx (xx.x)	xx (xx.x)	xx (xx.x)	xx (xx.x)	xx (xx.x)	xx (xx.x)	xx (xx.x)
Platelets ($1000/mm^3$)	xx (xx.x)	xx (xx.x)	xx (xx.x)	xx (xx.x)	xx (xx.x)	xx (xx.x)	xx (xx.x)	xx (xx.x)	xx (xx.x)	xx (xx.x)

PEEP - positive end-expiratory pressure; FiO_2 - fraction of inspired oxygen; PaO_2 - partial pressure of arterial oxygen; $PaCO_2$ - partial pressure of arterial carbon dioxide; SaO_2 - arterial oxy-hemoglobin saturation RASS - Richmond Agitation-Sedation Scale.

Efficacy outcomes

We will evaluate the effect of the ARDSNet strategy versus the STAMINA strategy on the primary outcome from a mixed ordinal model adjusted for age, COVID-19 diagnosis, baseline ventilatory ratio and PEEP at randomization, considering center as random effect in the intercept. Results will be reported as proportional odds ratios with a 95% confidence interval. It is suggested that the adjustment of the ordinal model in the R software is done with functions from the rms package.[4]

For the binary secondary outcomes (ICU and hospital mortality within 90 days; and need for rescue therapies: extracorporeal circulation, recruitment maneuver, inhaled nitric oxide), we will perform the comparison using mixed logistic regression models considering the random effect of the intercept and adjusted for the same variables considered in the primary outcome. Results will be presented using odds ratio.

For the continuous outcomes of interest (ICU length of stay, hospital length of stay, ICU-free days up to 28 days after randomization), we will use mixed generalized regression models considering the distribution that best fits the data (Poisson, Gamma, normal-inverse, etc.), considering the random effect of the intercept and adjusted for the same variables considered in the primary outcome. The results will present the mean difference with respective 95% confidence intervals.

The choice of data distribution chosen for the models will be evaluated through diagnostic graphs (eg: quantile-quantile and standardized residuals versus fitted data).

It is intended to present the results of the efficacy endpoints as described in table 5S below.

Table 5S - Efficacy outcomes assessment

Outcomes	STAMINA (n = 250)	ARDSNet (n = 250)	Effect estimation type	Effect estimate (95% CI)	p value
Primary outcome					
Ventilation-free days from, mean (SD)	xx (xx.x)	xx (xx.x)	ORp[1]	xx (xx-xx)	.xx
Median (IQR)	xx (xx-xx)	xx (xx-xx)		xx (xx-xx)	.xx
Secondary outcomes					
Death, No. of events/total (%)					
ICU	xx (xx.x)	xx (xx.x)	OR[2]	xx (xx-xx)	.xx
Hospital	xx (xx.x)	xx (xx.x)	OR	xx (xx-xx)	.xx
Rescue therapies, No. (%)					
Extracorporeal membrane oxygenation (ECMO)	xx (xx.x)	xx (xx.x)	OR	xx (xx-xx)	.xx
Recruitment maneuver	xx (xx.x)	xx (xx.x)	OR	xx (xx-xx)	.xx
Inhaled nitric oxide	xx (xx.x)	xx (xx.x)	OR	xx (xx-xx)	.xx
Exploratory outcomes					
Length of stay (days)					
ICU, mean (SD)	xx (xx.x)	xx (xx.x)	MD[3]	xx (xx-xx)	.xx
Median (IQR)	xx (xx-xx)	xx (xx-xx)		xx (xx-xx)	
Hospital, mean (SD)	xx (xx.x)	xx (xx.x)	MD	xx (xx-xx)	.xx
Median (IQR)	xx (xx-xx)	xx (xx-xx)		xx (xx-xx)	
ICU-free days in 28 days, mean (SD)	xx (xx.x)	xx (xx.x)	MD	xx (xx-xx)	.xx
Median (IQR)	xx (xx-xx)	xx (xx-xx)		xx (xx-xx)	
Oxygenation (PaO_2/FiO_2), mean (SD)	xx (xx.x)	xx (xx.x)	MD	xx (xx-xx)	.xx
Median (IQR)					
Ventilatory ratio, mean (SD)	xx (xx-xx)	xx (xx-xx)	MD	xx (xx-xx)	.xx
Median (IQR)					
Driving pressure (cmH_2O) mean (SD)	xx (xx-xx)	xx (xx-xx)	MD	xx (xx-xx)	.xx
Median (IQR)					
Oxygenation index, mean (SD)	xx (xx.x)	xx (xx.x)	MD	xx (xx-xx)	.xx
Median (IQR)					
Safety outcomes, No./total No. (%)					
Barotrauma	xx/xx (xx.x)	xx/xx (xx.x)	OR	xx (xx-xx)	.xx
Other - Serious adverse events	xx/xx (xx.x)	xx/xx (xx.x)	OR	xx (xx-xx)	.xx

ICU - intensive care unit; PaO_2 - partial pressure of arterial oxygen; FiO_2 - fraction of inspired oxygen. [1] Proportional odds ratio; [2] odds ratio; [3] mean difference.

The exploratory outcomes of table 5S help to explain the mechanism of possible intervention effect. The intervention is expected to reduce the mean driving pressure during the first three days compared to the control, and consequently improve other respiratory parameters such as the PaO_2/FiO_2 ratio and the oxygenation index.

Comparisons between these continuous parameters will be made by generalized mixed linear regression

models considering the distribution that best fits the data, considering the random effect of the research subject in the intercept and the days as fixed factors interacting with the group. The models will be fitted by the same adjustment covariates as the primary model.

Barotrauma and serious adverse event rate will be presented accumulated over the 3 days and compared from mixed logistic regression models with the center as a random effect intercept.

Assessments of adverse events and serious adverse events will be described using absolute and relative frequency tables and compared between patients by chi-square tests.

The distribution of serious adverse event results will also be described using frequency tables. The study pays special attention to the occurrence of barotraumas (subcutaneous emphysema, pneumothorax and pneumomediastinum, pneumatocele). Table 5S will describe the occurrence of these events during the intervention period, but the occurrence of these events during hospitalization will be presented next to the table of adverse events (Table 6S).

Table 6S - Serious adverse event during hospitalization

	STAMINA	ARDSNET
Serious adverse events (SAE), n	x/x (xx.x)	x/x (xx.x)
Patients with at least one serious adverse events, n/N (%)		
Type - SAE, n/N (%)		
Death	x/x (xx.x)	x/x (xx.x)
Need for hospitalization	x/x (xx.x)	x/x (xx.x)
Life threatining	x/x (xx.x)	x/x (xx.x)
Increase length of stay	x/x (xx.x)	x/x (xx.x)
Adverse events of interest n/N(%)		
Barotrauma (pneumothorax/pneumomediastinum)	x/x (xx.x)	x/x (xx.x)
Causal relationship of SAE with Study intervention, n/No. (%)		
Not related		
Possibly related	x/x (xx.x)	x/x (xx.x)
Probably related	x/x (xx.x)	x/x (xx.x)
Definitely related	x/x (xx.x)	x/x (xx.x)

Sensitivity analysis

All efficacy analyzes will also be performed on the population that followed the protocol without deviations, that is, they have all the inclusion and exclusion criteria and performed the interventions as stated in the protocol, without interrupting the maneuvers for reasons not specified in the protocol.

Subgroup analysis

We will analyze the effect of primary outcome interventions on the following interest groups:

1. Patients with and without a diagnosis of COVID-19 (with confirmation by RT-PCR test)
2. Patients with driving pressure above or below 15cmH_2O before randomization.

The subgroup analysis will be carried out with the inclusion of the interaction effect between the variables of interest and the groups in the primary proportional odds model considering the same adjustment variables and the random effect of center in the intercept.

Data imputation

The study will be carried out in the ICU and, therefore, the loss of follow-up and data should be minimal. It is not intended to carry out any data imputation, whether of outcome or base data.

REFERENCES

1. R Core Team. R: A language and environment for statistical computing. Vienna, Austria: R Foundation for Statistical Computing; 2021. Available from: URL https://www.R-project.org/.
2. Tomazini BM, Maia IS, Cavalcanti AB, Berwanger O, Rosa RG, Veiga VC, Avezum A, Lopes RD, Bueno FR, Silva MV, Baldassare FP, Costa EL, Moura RA, Honorato MO, Costa AN, Damiani LP, Lisboa T, Kawano-Dourado L, Zampieri FG, Olivato GB, Righy C, Amendola CP, Roepke RM, Freitas DH, Forte DN, Freitas FG, Fernandes CC, Melro LM, Junior GF, Morais DC, Zung S, Machado FR, Azevedo LC; COALITION COVID-19 Brazil III Investigators. Effect of dexamethasone on days alive and ventilator-free in patients with moderate or severe acute respiratory distress syndrome and COVID-19: the CoDEX randomized clinical trial. JAMA. 2020;324(13):1307-16.
3. Haybittle JL. Repeated assessment of results in clinical trials of cancer treatment. Br J Radiol. 1971;44(526):793-7.
4. Harrell Jr F. rms: Regression Modeling Strategies. R package version 6.7-1. 2023. Available from: https://CRAN.R-project.org/package=rms

APPENDIX 3 - DATA MONITORING COMMITTEE (DMC) CHARTER FOR THE STAMINA TRIAL

January, 2022

Introduction

This Charter is for the Data Monitoring Committee (DMC) for the Prospective, randomized, controlled trial comparing different ventilatory strategies in patients with acute respiratory distress syndrome due to community-acquired pneumonia: STAMINA.

The Charter will define the primary responsibilities of the DMC, its membership, and the purpose and timing of its meetings. The charter will also provide the statistical monitoring guidelines to be implemented by the DMC, and an outline of the content of the meetings (both open and closed).

Responsibilities of the DMC

1. To help ensure the safety of patients in the trial by protecting them from avoidable harm.
2. To provide the Steering Committee with advice about the conduct of the trial and the integrity of the data, so as to protect the validity and scientific credibility of the trial. In this regard, the DMC may provide suggestions regarding selection, recruitment and retention of participants; study interventions; adherence to protocol-specified regimens; and the procedures for data management and quality control. However, the DMC will have only a limited role on this issue because their detailed review of trial progress will occur only infrequently.
3. To evaluate interim analyses and judge efficacy, harm, and net clinical effect.

DMC composition

The DMC Chair, Rinaldo Bellomo from Australia, has been invited by the Coordinating Centre. DMC members have been selected by the DMC chair in collaboration with the steering committee for their trial experience plus expertise with intensive care medicine and/or statistics. DMC members are Professors Paul Young, MD, intensivist and clinical trialist, from New Zealand, and Prof. Michael Baileys, statistician, from Australia.

Conflict of interest

DMC members will disclose to the DMC Chair any present conflicts that they consider relevant, and any new conflicts that arise as the study proceeds. The DMC chair will disclose his conflicts, and any conflicts that arise, to the Chair of the Steering Committee, who will judge whether conflicts are of concern.

The Steering Committee Chair and DMC Chair have reviewed conflicts and determined that current conflicts will not compromise the DMC members from executing their role disinterestedly.

Meetings

Frequency of meetings

1. An initial meeting between the DMC and the STAMINA steering committee early in the trial is planned. Afterwards, DMC will meet to review interim analyses (see “Interim analyses” below).
2. The DMC Chair may request a full meeting of the committee at any time. Conversely, the steering committee may also propose a meeting with the DMC if necessary.

Structure of meetings

1. The initial meeting, with the purpose to finalize the DMC charter, will be open to the steering committee. This meeting may be replaced by online e-mail exchanges if all members of the DMC agree.
2. Meetings to review interim analyses will have the following structure:
    a. First, an open session with the principal investigator (PI), members of the steering committee, and members of coordinating centre (all of whom remain blinded to treatment specific data) to review accrual, data timeliness and quality, completeness of follow-up, problems with specific centres, and any proposals for changes in the study protocol or study duration. In addition, the PI will be responsible for reporting any new external evidence (especially results from other relevant ongoing trials) that bear on the conduct of the trial. No unblinded information will be revealed during this session.
    b. Second, a closed session (PI, steering committee, and coordinating centre members leave) between the DMC and the unblinded independent statistician(s) to review unblinded data on efficacy and safety, and the status of statistical monitoring boundaries.

c. Third, an optional executive session may be held with only DMC members present.

d. Lastly, an open session between the DMC and the blinded PI and steering committee will be held to deliver and discuss the DMC comments and recommendations and to decide on the timing of the next meeting. This session may be held by telephone or tele/videoconference.

Minutes

The Chair, or someone delegated by the Chair, will take minutes at closed sessions. The PI, or someone delegated by the PI, will take minutes at open meetings. The DMC Statistician will be responsible for archiving the closed session minutes. These will be considered confidential and should be available only for DMC members until the end of the trial. After each meeting the DMC Chair will provide the PI with a letter stating the general outcome of this meeting and suggested changes to the trial conduct. For example, this letter may simply contain the statement that the trial should continue as planned.

Decisions about stopping the trial

Based on interim analyses, and, possibly, on external evidence, the Data Monitoring Committee shall decide whether there is evidence beyond a reasonable doubt that the experimental treatment is deleterious for all patients or for any subgroup. The DMC may also decide that the accumulating data provides overwhelmingly convincing evidence that the experimental is superior to control treatment and recommend stopping the study for efficacy.

In the event that the DMC recommends the trial comparison be stopped, they will immediately notify the PI. The DMC will explain the basis of their recommendation to the steering committee and discuss the results together.

If the steering committee, and DMC agree as to the course of action, that is, to stop the trial comparison early, plans will be put into operation for the orderly conclusion of the trial, notification of study patients and sites, and dissemination of the results.

In the unlikely event that the DMC and steering committee members disagree about the proper course of action, the steering committee and DMC will make every attempt to reach a consensus through discussions. If, despite best efforts, significant differences of opinion persist, then additional input from individuals (selected by mutual agreement) will be sought. Every attempt will be made to reach a consensus through this process.

Interim analyses

Role of the coordinating center and independent statistician

Every effort will be made by the Coordinating Centre to provide the data for interim analyses to the DMC without delay, in order to ensure the safety of patients. A blind statistician will conduct the interim analyses and present them to the Data Monitoring Committee. Results of interim analyses must not be presented to the steering committee, members of study office or any investigators.

Frequency of interim analysis

Two interim analyses are planned:

1. Once 100 patients complete 28 days of follow-up, preliminary safety data will be shared with the DMC, along with treatment adherence results (Control/ARDSNET and Intervention). In this interim analysis, efficacy outcomes will not be evaluated, and therefore, the study cannot be interrupted for benefit.

2. Once 200 patients complete 28 days of follow-up, the DMC will assess the results of all outcomes in a non-blind manner (safety and efficacy). In light of the review of adverse events and external evidence, the Data Monitoring Committee should assess whether there is evidence beyond reasonable doubt that one of the interventions is clearly contraindicated for all patients or some subgroup. For interruptions for efficacy (superiority) or safety based on the ventilator-free days (primary outcome), it is suggested that the DMC consider the Haybittle-Peto criterion (P value <0.001).[1] As the Haybittle-Peto criterion[1] is rigorous, there is practically no consumption of type I error, and thus the final evaluation is maintained considering a significance level fixed at 5%.

Stopping boundaries

The DMC will utilize statistical monitoring boundaries as proposed in this charter and external evidence. These boundaries will be considered guidelines, not rules. Any DMC recommendation should be based on the pattern of all outcomes (efficacy and safety) within the trial and the totality of evidence in existence.

Stopping for safety

If any of the interim analyses shows that the experimental intervention compared to control is associated with a higher mortality in 28 days with a two-sided p value < 0.01, this

will trigger DMC discussions about stopping the comparison for harm. Serious Adverse Events. Serious adverse events which are study related according to the site investigators should be urgently reported (within 24 hours from the onset of the event) to the coordinating center. Those events will be forwarded to the DMC members. A serious adverse event directly related to the study is defined as any event meeting the three following criteria:

1. Any fatal or life-threatening event (immediate risk of death), or any event that causes sequelae or permanent disability, or that extends hospitalization;
2. Occurrence of barotrauma (subcutaneous emphysema, pneumothorax, pneumatocele or pneumomediastinum);
3. Other serious adverse events possibly related to mechanical ventilation.

Publication policy

The PI will provide the DMC with a copy of the intended main trial results publication 14 days prior to the intended submission, in order to allow the DMC to review the intended publication and provide input. The DMC will recommend any changes to the publication it reasonably believes are necessary for scientific purposes. The PI and Coordinating Centre agree to thoroughly consider the implementation of all such recommended changes. Notwithstanding the above, the final decision regarding the content of any publication shall be that of the Coordinating Centre.

* STAMINA Study Group Investigators

Israel Silva Maia, Fernando Azevedo Medrado Jr, Lucas Tramujas, Bruno Martins Tomazini, Júlia Souza Oliveira, Erica Regina Ribeiro Sady, Letícia Galvão Barbante, Marina Lazzari Nicola, Rodrigo Magalhães Gurgel, Lucas Petri Damiani, Karina Leal Negrelli, Tamiris Abait Miranda, Eliana Santucci, Nanci Valeis, Ligia Nasi Laranjeira, Glauco Adrieno Westphal, Ruthy Perotto Fernandes, Cássio Luis Zandonai, Mariangela Pimentel Pincelli, Rodrigo Cruvinel Figueiredo, Cíntia Loss Sartori Bustamante, Luiz Fernando Norbin, Emerson Boschi, Rafael Lessa, Marcelo Pereira Romano, Mieko Cláudia Miura, Meton Soares de Alencar Filho, Vicente Cés de Souza Dantas, Priscilla Alves Barreto, Mauro Esteves Hernandes, Cintia Magalhães Carvalho Grion, Alexandre Sanches Laranjeira, Ana Luiza Mezzaroba, Marina Bahl, Ana Carolina Starke, Rodrigo Santos Biondi, Felipe Dal-Pizzol, Eliana Bernadete Caser, Marlus Muri Thompson, Andrea Allegrini Padial, Viviane Cordeiro Veiga, Rodrigo Thot Leite, Gustavo Araújo, Mário Guimarães, Priscilla de Aquino Martins, Fábio Holanda Lacerda, Conrado Roberto Hoffmann Filho, Livia Melro, Eduardo Pacheco, Gustavo Adolfo Ospina-Táscon, Juliana Carvalho Ferreira, Fabricio Jocundo Calado Freires, Flávia Ribeiro Machado, Alexandre Biasi Cavalcanti, Fernando Godinho Zampieri

REFERENCE

1. Haybittle JL. Repeated assessment of results in clinical trials of cancer treatment. Br J Radiol. 1971;44(526):793-7.
