# Supplementary material for: Prospective, randomized, controlled trial assessing the effects of a driving pressure–limiting strategy for patients with acute respiratory distress syndrome due to community-acquired pneumonia (STAMINA trial): protocol and statistical analysis plan
Source: Crit Care Sci. 2024 Apr 26;36:e20240210en. doi: 10.62675/2965-2774.20240210-en (PMC11098077; doi:10.62675/2965-2774.20240210-en)
Supplement: Translation

# Translation: Estudo randomizado prospectivo e controlado de avaliação dos efeitos de uma estratégia de limitação da *driving pressure* em pacientes com síndrome do desconforto respiratório agudo devido à pneumonia adquirida na comunidade (estudo STAMINA): protocolo e plano de análise estatística

**DOI:** 10.62675/2965-2774.20240210-pt

**Authors:** STAMINA Study Group Investigators, Israel Silva Maia, Fernando Azevedo Medrado, Lucas Tramujas, Bruno Martins Tomazini, Júlia Souza Oliveira, Erica Regina Ribeiro Sady, Letícia Galvão Barbante, Marina Lazzari Nicola, Rodrigo Magalhães Gurgel, Lucas Petri Damiani, Karina Leal Negrelli, Tamiris Abait Miranda, Eliana Santucci, Nanci Valeis, Ligia Nasi Laranjeira, Glauco Adrieno Westphal, Ruthy Perotto Fernandes, Cássio Luis Zandonai, Mariangela Pimentel Pincelli, Rodrigo Cruvinel Figueiredo, Cíntia Loss Sartori Bustamante, Luiz Fernando Norbin, Emerson Boschi, Rafael Lessa, Marcelo Pereira Romano, Mieko Cláudia Miura, Meton Soares de Alencar, Vicente Cés de Souza Dantas, Priscilla Alves Barreto, Mauro Esteves Hernandes, Cintia Magalhães Carvalho Grion, Alexandre Sanches Laranjeira, Ana Luiza Mezzaroba, Marina Bahl, Ana Carolina Starke, Rodrigo Santos Biondi, Felipe Dal-Pizzol, Eliana Bernadete Caser, Marlus Muri Thompson, Andrea Allegrini Padial, Viviane Cordeiro Veiga, Rodrigo Thot Leite, Gustavo Araújo, Mário Guimarães, Priscilla de Aquino Martins, Fábio Holanda Lacerda, Conrado Roberto Hoffmann, Livia Melro, Eduardo Pacheco, Gustavo Adolfo Ospina-Táscon, Juliana Carvalho Ferreira, Fabricio Jocundo Calado Freires, Flávia Ribeiro Machado, Alexandre Biasi Cavalcanti, Fernando Godinho Zampieri

**Affiliations:** 1 São Paulo SP Brasil Instituto de Pesquisa, Hcor-Hospital do Coração - São Paulo (SP), Brasil.; 2 São Paulo SP Brasil Departamento de Anestesiologia, Dor e Terapia Intensiva, Universidade de São Paulo - São Paulo (SP), Brasil.; 3 São Paulo SP Brasil Brazilian Research in Intensive Care Network (BRICNet) - São Paulo (SP), Brasil.; 4 Joinville SC Brasil Centro Hospitalar Unimed Joinville - Joinville (SC), Brasil.; 5 Florianópolis SC Brasil Hospital Nereu Ramos - Florianópolis (SC), Brasil.; 6 Colatina ES Brasil Hospital e Maternidade São José - Colatina (ES), Brasil.; 7 Linhares ES Brasil Linhares Medical Center - Linhares (ES), Brasil.; 8 Caxias do Sul RS Brasil Hospital Geral de Caxias do Sul - Caxias do Sul (RS), Brasil.; 9 São Paulo SP Brasil Hcor-Hospital do Coração - São Paulo (SP), Brasil.; 10 Barbalha CE Brasil Hospital São Vicente de Paulo - Barbalha (CE), Brasil.; 11 Rio de Janeiro RJ Brasil Hospital Marcílio Dias - Rio de Janeiro (RJ), Brasil.; 12 Votuporanga SP Brasil Santa Casa de Votuporanga - Votuporanga (SP), Brasil.; 13 Londrina PR Brasil Hospital Universitário, Universidade Estadual de Londrina - Londrina (PR), Brasil.; 14 Londrina PR Brasil Hospital Araucária de Londrina - Londrina (PR), Brasil.; 15 Florianópolis SC Brasil Hospital Universitário, Universidade Federal de Santa Catarina - Florianópolis (SC), Brasil.; 16 Brasília DF Brasil Hospital Brasília - Brasília (DF), Brasil.; 17 Criciúma SC Brasil Hospital São José - Criciúma (SC), Brasil.; 18 Vitória SC Brasil Hospital Unimed Vitória - Vitória (SC), Brasil.; 19 Cachoeiro de Itapemirim ES Brasil Hospital Evangélico de Cachoeiro de Itapemirim - Cachoeiro de Itapemirim (ES), Brasil.; 20 Florianópolis SC Brasil Instituto Baía Sul - Florianópolis (SC), Brasil.; 21 São Paulo SP Brasil BP - A Beneficência Portuguesa de São Paulo - São Paulo (SP), Brasil.; 22 Florianópolis SC Brasil Imperial Hospital de Caridade - Florianópolis (SC), Brasil.; 23 Barretos SP Brasil Santa Casa de Misericórdia de Barretos - Barretos (SP), Brasil.; 24 Serra ES Brasil Hospital Estadual Dr. Jayme Santos Neves - Serra (ES), Brasil.; 25 Fortaleza CE Brasil Hospital Otoclínica - Fortaleza (CE), Brasil.; 26 Joinville SC Brasil Hospital Regional Hans Dieter Schmidt - Joinville (SC), Brasil.; 27 São Paulo SP Brasil Hospital Samaritano, São Paulo (SP), Brasil.; 28 São Paulo SP Brasil Hospital Sepaco - São Paulo (SP), Brasil.; 29 Colombia CO Colombia Fundación Valle del Lili - Universidad ICESI - Colombia, CO.; 30 São Paulo SP Brasil Departamento de Pneumologia, Instituto do Coração, Hospital das Clínicas, Faculdade de Medicina, Universidade de São Paulo - São Paulo (SP), Brasil.; 31 São Paulo SP Brasil Departamento de Anestesiologia, Dor e Medicina Intensiva, Universidade Federal de São Paulo - São Paulo (SP), Brasil.; 32 Alberta Canada Department of Critical Care Medicine, Faculty of Medicine and Dentistry, University of Alberta and Alberta Health Services - Edmonton, Alberta, Canada.

**Keywords:** Oxigenação por membrana extracorpórea, Síndrome do desconforto respiratório, Respiração com pressão positiva, Respiração artificiai, Lesão pulmonar induzida por ventilação mecânica, Pneumonia

## Abstract

**Contexto:**

Em estudos observacionais sobre a síndrome do desconforto respiratório agudo, sugeriu-se que a *driving pressure* é o principal fator de lesão pulmonar induzida por ventilador e de mortalidade. Não está claro se uma estratégia de limitação da *driving pressure* pode melhorar os desfechos clínicos.

**Objetivo:**

Descrever o protocolo e o plano de análise estatística que serão usados para testar se uma estratégia de limitação da *driving pressure* envolvendo a titulação da pressão positiva expiratória final de acordo com a melhor complacência respiratória e a redução do volume corrente é superior a uma estratégia padrão envolvendo o uso da tabela de pressão positiva expiratória final baixa do protocolo ARDSNet, em termos de aumento do número de dias sem ventilador em pacientes com síndrome do desconforto respiratório agudo devido à pneumonia adquirida na comunidade.

**Métodos:**

O estudo STAMINA (*ventilator STrAtegy for coMmunIty acquired pNeumoniA*) é randomizado, multicêntrico e aberto e compara uma estratégia de limitação da *driving pressure* com a tabela de pressão positiva expiratória final baixa do protocolo ARDSnet em pacientes com síndrome do desconforto respiratório agudo moderada a grave devido à pneumonia adquirida na comunidade internados em unidades de terapia intensiva. Esperamos recrutar 500 pacientes de 20 unidades de terapia intensiva brasileiras e duas colombianas. Eles serão randomizados para um grupo da estratégia de limitação da *driving pressure* ou para um grupo de estratégia padrão usando a tabela de pressão positiva expiratória final baixa do protocolo ARDSnet. No grupo da estratégia de limitação da *driving pressure*, a pressão positiva expiratória final será titulada de acordo com a melhor complacência do sistema respiratório.

**Desfechos:**

O desfecho primário é o número de dias sem ventilador em 28 dias. Os desfechos secundários são a mortalidade hospitalar e na unidade de terapia intensiva e a necessidade de terapias de resgate, como suporte de vida extracorpóreo, manobras de recrutamento e óxido nítrico inalado.

**Conclusão:**

O STAMINA foi projetado para fornecer evidências sobre se uma estratégia de limitação da *driving pressure* é superior à estratégia da tabela de pressão positiva expiratória final baixa do protocolo ARDSnet para aumentar o número de dias sem ventilador em 28 dias em pacientes com síndrome do desconforto respiratório agudo moderada a grave. Aqui, descrevemos a justificativa, o desenho e o status do estudo.

## INTRODUÇÃO

Apesar dos recentes avanços na assistência e no manejo de pacientes ventilados, a mortalidade devido à síndrome do desconforto respiratório agudo (SDRA) continua alta.^(1)^ A gravidade da SDRA de etiologia respiratória ficou especialmente evidente durante a pandemia da doença pelo coronavírus 2019 (COVID-19), durante a qual a mortalidade de pacientes ventilados ultrapassou 50%.^(2^^,^^3)^ São necessárias estratégias que melhorem a assistência a esses pacientes para diminuir a morbidade e a mortalidade.

O estiramento cíclico do parênquima pulmonar é um dos principais contribuintes para a lesão pulmonar induzida pela ventilação (LPIV) e varia conforme o volume de repouso reduzido do pulmão aerado em pacientes com SDRA,^(4)^ que, por sua vez, está fortemente correlacionado com a complacência do sistema respiratório. A *driving pressure* (DP) é uma variável facilmente mensurável, que é igual ao volume corrente dividido pela complacência do sistema respiratório. Em virtude dessas propriedades, propôs-se que a DP seja um mediador-chave da LPIV.^(5)^ A associação entre a DP e a mortalidade durante a ventilação mecânica (VM) é apoiada por dados observacionais.^(4^^,^^6)^ No entanto, atualmente não se sabe se uma estratégia de tratamento que limita a DP seria superior a uma estratégia de ventilação convencional, em termos de resultados clínicos.^(7)^ Estratégias eficazes para reduzir a DP incluem a melhor titulação da pressão positiva expiratória final (PEEP) e um volume corrente menor. A estratégia padrão de manejo da PEEP envolve o uso arbitrário da PEEP segundo a necessidade de uma fração inspirada de oxigênio (FiO_2_; "tabela PEEP").^(8^^,^^9)^ No entanto, ela pode se basear na melhor complacência do sistema respiratório, que é inversamente proporcional à DP.^(5)^ Ao ajustar gradualmente os níveis de PEEP enquanto monitoram as alterações na complacência, os médicos podem determinar a configuração ideal de PEEP que minimiza a DP.^(10^^,^^11)^ Além disso, a redução do volume corrente abaixo do padrão de 6mL/kg pode diminuir ainda mais a DP. A implementação dessas medidas em pacientes com SDRA permite uma abordagem personalizada do manejo do ventilador, reduzindo a DP e, possivelmente, mitigando a LPIV.

O objetivo deste estudo foi avaliar se uma estratégia de VM focada na redução da DP pode melhorar os resultados clínicos em comparação com a estratégia da tabela de PEEP baixa do protocolo ARDSnet em pacientes com SDRA moderada a grave secundária à pneumonia adquirida na comunidade (PAC). A PAC é responsável por 50% dos casos de SDRA em alguns estudos clínicos.^(1^^,^^12)^ A inclusão apenas de pacientes com PAC pode melhorar a taxa de recrutamento, especialmente durante pandemias, e diminuir a chance de encontrar efeitos heterogêneos do tratamento entre as etiologias da SDRA.

Este estudo está sendo conduzido para avaliar o impacto dessa nova abordagem nos principais desfechos clínicos, como dias sem ventilador e melhora geral nos parâmetros respiratórios. Ao comparar esses resultados entre as duas estratégias de ventilação, pretendemos determinar se uma abordagem focada na DP pode proporcionar benefícios superiores em relação a resultados clinicamente importantes e, possivelmente, abrir caminho para o manejo ventilatório otimizado em pacientes com SDRA.

## MÉTODOS

### Desenho do estudo

O estudo STAMINA (*ventilator STrAtegy for coMmunIty acquired pNeumoniA*) é randomizado, multicêntrico e aberto e comparará duas estratégias ventilatórias: uma de limitação da DP *versus* uma padrão (tabela de PEEP baixa do protocolo ARDSNet) em pacientes com SDRA moderada a grave secundária à PAC que estão hospitalizados em unidades de terapia intensiva (UTIs). Os pacientes aptos a participar receberão uma dessas estratégias. O protocolo deste estudo é relatado seguindo as recomendações da Declaração SPIRIT 2013.^(13)^

### Objetivos e desfechos

Avaliar se uma estratégia de limitação da DP para titular a PEEP conforme a melhor adesão é superior em termos de aumento do número de dias sem ventilador em comparação com a estratégia padrão que usa uma tabela de PEEP baixa. A tabela 1 apresenta os desfechos primários, secundários, exploratórios e de segurança.

**Tabela 1:** Desfechos

Desfechos	
Primários	Dias sem ventilador em 28 dias a partir da randomização ou até a alta hospitalar, medidos da seguinte forma:
	D = 0 (se o paciente morrer dentro de 28 dias no hospital ou permanecer em suporte respiratório com VM ≥ 28 dias)
	D = 28 - x (se o paciente receber alta hospitalar em < 28 dias, em que x representa o número de dias com VM durante a hospitalização)
	O número de dias em VM será contado como cada dia em que o paciente passou pelo menos 12 horas em VM. Se houver uma interrupção da VM seguida de reinício dentro de 48 horas (falha na extubação), o período inteiro será computado como um único período. Para pacientes traqueostomizados, o mesmo critério é válido. É computado 1 dia de ventilação sempre que o paciente persistir por mais de 12 horas em ventilação
Secundários	1. Mortalidade intra-hospitalar em 90 dias
	2. Mortalidade na UTI em 90 dias
	3. Necessidade de terapias de resgate (circulação extracorpórea, manobra de recrutamento fora do protocolo do estudo, óxido nítrico inalado) em 28 dias
Exploratórios	1. Oxigenação, medida por PaO_2_/FiO_2_ durante os primeiros 3 dias
	2. *Driving pressure* durante os primeiros 3 dias
	3. Taxa ventilatória, definida como PaCO_2_ multiplicada pela ventilação por minuto dividida por 100 × peso predito em quilogramas × 37,5, medida nos primeiros 3 dias
	4. Índice de oxigenação, definido por PMVA × FiO_2_ × 100 ÷ PaO_2_, medido nos primeiros 3 dias
	5. Potência mecânica, definida pela transferência de energia (0,098) × frequência respiratória × volume corrente × [pressão de pico das vias aéreas - 0,5 × (pressão de platô - PEEP)], medida nos primeiros 3 dias
	6. Dias fora da UTI em 28 dias
	7. Tempo de internação na UTI
	8. Tempo de internação hospitalar
De segurança	1. Ocorrência de barotrauma (enfisema subcutâneo, pneumotórax ou pneumomediastino)
	2. Outros eventos adversos graves possivelmente relacionados à VM

VM - ventilação mecânica; UTI - unidade de terapia intensiva; PaO_2_ - pressão parcial de oxigênio; FiO_2_ - fração inspirada de oxigênio; PaCO_2_ - pressão parcial de dióxido de carbono arterial; PMVA - pressão média das vias aéreas; PEEP - pressão positiva expiratória final.

### Locais e recrutamento

Pelo menos 20 UTIs brasileiras e duas colombianas. A taxa de recrutamento esperada é de um paciente/mês/local. A duração planejada do estudo é estimada em dois anos e meio.

### Critérios de inclusão e exclusão

Os pacientes devem ter ≥ 18 anos de idade, apresentar síndrome respiratória aguda moderada ou grave devido à PAC e estar recebendo VM. Os critérios de inclusão estão detalhados na tabela 2. O fluxograma do paciente está detalhado na figura 1.

**Tabela 2:** Critérios de seleção

Critérios de inclusão	Critérios de exclusão
Pacientes com PAC em VM invasiva (a PAC deve ter sido a causa do início da VM) Infiltrado bilateral agudo de origem não exclusivamente cardiogênica, de acordo com o julgamento do médico assistente Um dos itens abaixo: FiO_2_ acima de 50% com PEEP de pelo menos 8cmH_2_O para manter a saturação acima de 93% OU PaO_2_/FiO_2_ < 200 com PEEP > 5cmH_2_O	Pacientes com critérios de inclusão por mais de 36 horas Hipertensão intracraniana ou doença neurológica aguda (acidente vascular cerebral, hemorragia subaracnoidea) Recusa do representante legal Pacientes com menos de 18 anos de idade Pacientes considerados não candidatos a medidas de suporte invasivo completo no momento da randomização, ou seja, pacientes que tenham alguma indicação clara, no momento da randomização, para não instituir outras medidas de suporte invasivas Paciente com fístula brônquica ou barotrauma Pacientes com histórico de uso domiciliar de oxigênio por doença respiratória crônica

PAC - pneumonia adquirida na comunidade; VM - ventilação mecânica; FiO_2_ - fração inspirada de oxigênio; PEEP - pressão positiva expiratória final; PaO_2_ - pressão parcial de oxigênio.

Nota: O critério de inclusão 3a pressupõe a necessidade desses parâmetros para manter pelo menos 93% de saturação de oxigênio. Se o paciente for admitido com fração inspirada de oxigênio alta e/ou pressão positiva expiratória final alta, recomenda-se, se possível, tentar reduzir os parâmetros ventilatórios (fração inspirada de oxigênio e/ou pressão positiva expiratória final) para avaliar a resposta da saturação de oxigênio antes de considerar esse critério atendido.

**Figura 1:** Fluxograma do estudo.

PAC - pneumonia adquirida na comunidade; VM - ventilação mecânica; FiO_2_ - fração inspirada de oxigênio; PEEP - pressão positiva expiratória final; PaO_2_ - pressão parcial de oxigênio. TCLE - Termo de Consentimento Livre e Esclarecido. * O Termo de Consentimento Livre e Esclarecido pode ser assinado pelo parente mais próximo antes ou depois da randomização.

### Intervenções do estudo

As intervenções do estudo serão realizadas do Dia 0 (randomização) ao Dia 3. Os procedimentos de ventilação estão resumidos na tabela 3.

**Tabela 3:** Intervenções do estudo após a randomização de D0 a D3

Variável	Estratégia ARDSnet	Estratégia STAMINA
Ajustes basais		
Modo do ventilador	Controle de assistência de volume	Controle de assistência de volume
Pressão de platô (cmH_2_O)	≤ 30	≤ 30
Volume corrente inicial (mL/kg de peso corporal previsto)	6 ou menor para manter a Pplat ≤ 30cmH_2_O	6 ou menor para manter a Pplat ≤ 30cmH_2_O
Configuração da frequência respiratória necessária para atingir pH ≥ 7,2	Até 35 respirações/minuto	Até 35 respirações/minuto
Meta de oxigenação	SpO_2_ 90 - 94% e PaO_2_ ≥ 60mmHg	SpO_2_ 90 - 94% e PaO_2_ ≥ 60mmHg
Formato da onda de fluxo	Descendente ou quadrada	Descendente ou quadrada
Pausa inspiratória	Máximo de 0,5 segundo	Máximo de 0,5 segundo
Fluxo (L/minuto)	30 - 60	30 - 60
I:E	1:1 - 1:2	1:1 - 1:2
Ajustes de titulação da PEEP		
Titulação da PEEP*	Tabela de PEEP baixa - FiO_2_	Fase incremental: até PEEP de 20cmH_2_O Fase decrescente: redução da PEEP em etapas de 2cmH_2_O PEEP titulada pela melhor complacência estática do sistema respiratório
Critérios de suspensão da titulação da PEEP		Pressão de pico das vias aéreas ≥ 40cmH_2_O Pressão de platô ≥ 33cmH_2_O Arritmias cardíacas instáveis aumentam a dose de vasopressor > 20micg/minuto†
Fase decrescente		Pausa inspiratória de 2 segundos no final de cada etapa para medir o valor de complacência até PEEP de 8cmH_2_O‡ A melhor PEEP será o valor imediatamente anterior de uma queda na complacência de pelo menos 2mL/cmH_2_O em 1 diminuição ou de pelo menos 1mL/cmH_2_O em 2 diminuições sequenciais‡
*Driving pressure*	Não é permitido controlar	Controlado para manter ≤ 14
Ajustes adicionais	Se a pressão de platô for > 30cmH_2_O: – Redução do volume corrente até 4mL/kg de peso corporal previsto, seguida de redução da PEEP em etapas de 2cmH_2_O (se não for resolvido com a redução do volume corrente) – Se necessário, é permitido aumentar a FR em até 40 respirações/minuto se o pH for < 7,2 e a PaCO_2_ > 70mmHg com PEEP automática não superior a 2cmH_2_O	Se a DP for > 14 e/ou a pressão de platô for > 30: – Redução do volume corrente até 4mL/kg de peso corporal previsto, seguida de redução da PEEP em etapas de 2cmH_2_O (se não for resolvido com a redução do volume corrente) – Se necessário, é permitido aumentar a FR em até 40 respirações/minuto se o pH for < 7,2 e a PaCO_2_ > 70mmHg com PEEP automática não superior a 2cmH_2_O
Monitoramento da mecânica	Duas vezes por dia com intervalo de 8 horas a partir de cada avaliação	Duas vezes por dia com intervalo de 8 horas a partir de cada avaliação
Sedação	Sedação profunda com RASS -5 e bloqueio neuromuscular nas primeiras 72 horas se o paciente mantiver a ventilação controlada. Após esse período, sedação leve (RASS 0 a -2)	Sedação profunda com RASS -5 e bloqueio neuromuscular nas primeiras 72 horas, se o paciente mantiver a ventilação controlada. Após esse período, sedação leve (RASS 0 a -2)
Novo procedimento de titulação em menos de 24 horas	Pplat > 30cmH_2_O, mudança de posição corporal (decúbito ventral ou dorsal), despressurização do circuito	Pplat > 30cmH_2_O, DP > 14cmH_2_O, mudança de posição corporal (decúbito ventral ou dorsal), despressurização do circuito
Rotina de titulação	D0, D1, D2, D3 com 24 horas de intervalo	D0, D1, D2, D3 com 24 horas de intervalo
Desmame da PEEP após 24 horas com PaO_2_/FiO_2_ > 200	Diminuir 2cmH_2_O em intervalos de 8 horas	Diminuir 2cmH_2_O em intervalos de 8 horas
Desmame da ventilação	FiO_2_ ≤ 50% com PEEP ≤ 10cmH_2_O	FiO_2_ ≤ 50% com PEEP ≤ 10cmH_2_O

Pplat - pressão de platô; SpO_2_ - saturação de oxigênio; PaO_2_ - pressão parcial de oxigênio; I:E - relação entre a duração da inspiração e a duração da expiração; PEEP - pressão positiva expiratória final; FiO_2_ - fração inspirada de oxigênio; FR - frequência respiratória; PaCO_2_ - pressão parcial de dióxido de carbono arterial; DP - *driving pressure*; RASS - escala de agitação e sedação de Richmond.

O peso previsto deve ser calculado para todos os pacientes de acordo com a seguinte fórmula: homens: peso previsto (kg) = 50 + 2,3 {[altura (cm) × 0,394] - 60}, mulheres: peso previsto (kg) = 45,5 + 2,3 {[altura (cm) × 0,394] - 60}.

* No braço STAMINA, a manobra de titulação pode ser adiada em um determinado dia se o paciente tiver uma relação entre pressão parcial de oxigênio e fração inspirada de oxigênio acima de 200, a pressão de platô estiver abaixo de 30cmH_2_O, a *driving pressure* não for superior a 14 cmH_2_O e a pressão positiva expiratória final não for superior a 10cmH_2_O;

† se qualquer regra de critério de parada for atingida, a fase decremental começará a partir do valor da pressão positiva expiratória final em que a fase incremental parou;

‡ se houver um empate entre vários valores de pressão positiva expiratória final, será usado seu menor valor.

### Grupo intervenção – estratégia STAMINA

A estratégia STAMINA compreende uma estratégia de DP limitada que inclui a titulação da PEEP orientada pela melhor complacência do sistema respiratório. A PEEP é titulada da seguinte forma: inicialmente, é realizada uma fase incremental, aumentando a PEEP até 20cmH_2_O, respeitando os critérios de parada de segurança detalhados na tabela 3. No entanto, na fase decremental, a PEEP é reduzida em 2cmH_2_O a cada minuto e mantida no nível com a melhor complacência estática. Posteriormente, o volume corrente e/ou a PEEP podem ser ajustados para manter uma DP ≤ 14cmH_2_O e uma pressão de platô (Pplat) ≤ 30cmH_2_O. A figura 2 mostra uma representação esquemática.

**Figura 2:** Titulação da pressão positiva expiratória final - estratégia STAMINA.

Esta figura mostra a estratégia STAMINA usada para conduzir a titulação da pressão positiva expiratória final. A pressão positiva expiratória final foi inicialmente aumentada para 20cmH_2_O e, posteriormente, diminuída em 2cmH_2_O após 1 minuto em cada nível, até atingir 8cmH_2_O. Depois disso, a pressão positiva expiratória final ideal é determinada com base no valor que produz a melhor complacência estática do sistema respiratório.

PEEP - pressão positiva expiratória final.

### Grupo controle – estratégia ARDSnet

A PEEP e a FiO_2_ serão ajustadas por meio da tabela de PEEP baixa do protocolo ARDSNet (Tabela 4), para manter a saturação de oxigênio (SpO_2_) entre 90 e 94% ou pressão parcial de oxigênio (PaO_2_) ≥ 60mmHg. Posteriormente, o volume corrente e/ou a PEEP podem ser ajustados para manter a Pplat ≤ 30cmH_2_O, independentemente da DP.

**Tabela 4:** Tabela pressão positiva expiratória final baixa-fração inspirada de oxigênio

FiO_2_ (%)	30	40	40	50	50	60	70	70	70	80	90	90	90	100
PEEP (cmH_2_O)	5	5	8	8	10	10	10	12	14	14	14	16	18	18 - 24

FiO_2_ - fração inspirada de oxigênio; PEEP - pressão positiva expiratória final.

### Tamanho da amostra

Planejamos randomizar 500 pacientes. Esse número proporciona um poder de pelo menos 90% para detectar diferença de aproximadamente 3 dias sem VM e taxa de mortalidade 5% menor no grupo em tratamento.

Considerando os dados brutos da parte não relacionada à morte do desfecho composto (dias sem ventilação) e taxa de mortalidade de 60% no grupo controle do estudo CoDEX^(3)^ como referência, nossas simulações alcançaram média de 4,7 dias sem VM em 28 dias no grupo controle, com quartis [3,7 - 5,8] e desvio-padrão de 8,2, e média de 7,8 dias sem VM no grupo tratamento, com quartis [6,5 - 9,2] e desvio-padrão de 10,5.

As estimativas de poder foram obtidas a partir de 2.000 simulações de diferentes cenários de desfecho primário, considerando que a morte dentro de 28 dias implicaria em 0 dia sem VM, mesmo que o paciente estivesse sem VM por pelo menos 1 dia durante a hospitalização. A figura 3 mostra a distribuição cumulativa do desfecho primário de uma simulação, considerando o modelo nulo (o efeito no grupo tratamento foi idêntico ao do grupo controle).

**Figura 3:** Distribuição cumulativa para o número de dias sem ventilação mecânica em 28 dias. Cenário nulo.

### Recrutamento

Atualmente, o recrutamento de pacientes está em andamento em 29 UTIs em todo o Brasil e 2 na Colombia. Esses centros colaboram ativamente com a Rede Brasileira de Pesquisa em Terapia Intensiva (BRICNet), garantindo alto padrão de recrutamento de pacientes e dados de alta qualidade. A maioria desses centros está localizada nas Regiões Sul ou Sudeste do Brasil, onde esperamos uma maior incidência de PAC relacionada à maior densidade populacional associada a um ambiente de temperatura mais baixa durante o outono e o inverno.

### Randomização e ocultação de alocação

A lista de randomização será gerada eletronicamente por meio de um *software* apropriado. A randomização será realizada em blocos (tamanho de bloco variável) estratificados por centro e diagnóstico da COVID-19. A confidencialidade da lista de randomização será mantida por meio do sistema central de randomização automatizada via internet, que está disponível 24 horas por dia (RedCap). O grupo no qual o paciente será alocado só será divulgado após o registro da informação no sistema eletrônico, o que impedirá que o investigador e a equipe assistente prevejam o grupo de tratamento ao qual o paciente será alocado. O pesquisador deve acessar o *site* usado no estudo (RedCap) para alocar formalmente os pacientes nos diferentes grupos de tratamento.

### Cegamento

Não haverá cegamento, devido à natureza da intervenção, que impossibilitará o mascaramento dos pesquisadores, participantes e avaliadores de resultados. No entanto, os avaliadores dos desfechos só terão acesso a estes dados após o final do estudo.

### Sequência de tempo de coleta de dados

Os dados que serão coletados estão especificados na tabela 5, de acordo com a figura 4.

**Tabela 5:** Cronograma de coleta de dados

	D-1	D0	D1	D2	D3	D28	Alta da UTI	Alta hospitalar
Critérios de aptidão	X							
Randomização		X						
Termo de Consentimento Livre e Esclarecido	X							
Tempo entre a admissão à UTI e a randomização		X						
Tempo de VM antes da randomização		X						
Data de nascimento		X						
Parâmetros ventilatórios		X	X	X	X			
Gasometria arterial		X	X	X	X			
Sexo no nascimento	X							
Altura	X							
Comorbidades	X							
Etiologia da pneumonia	X							
Dose de vasopressor		X	X	X	X	X	X	
RASS na randomização		X						
Parâmetros SAPS 3		X						
Suporte ao protocolo: intervenções		X	X	X	X			
Posição em decúbito ventral		X	X	X	X		X	
Terapias de resgate		X	X	X	X		X	
Uso de vasopressores		X	X	X	X		X	
Creatinina	X	X	X	X	X			
Bilirrubina total	X	X	X	X	X			
Data e hora da extubação			X	X	X	X	X	
Data e hora da reintubação			X	X	X	X	X	
Estado vital na alta da UTI						X	X	
Data do óbito						X	X	X
Número de dias em uso de VM no hospital						X	X	X
Data da traqueostomia						X	X	X
Dados da alta da UTI							X	
Dados da alta hospitalar								X
Estado vital na alta hospitalar								X

UTI - unidade de terapia intensiva; VM - ventilação mecânica; RASS - escala de agitação e sedação de Richmond; SAPS 3 - *Simplified Acute Physiology Score 3*.

**Figura 4:** Linha do tempo do participante.

D - dia.

### Coleta e tratamento de dados

Faremos o resumo dos dados coletados rotineiramente na UTI por meio de um banco de dados digital de fácil acesso pela internet (RedCap).

Vários procedimentos garantirão a qualidade dos dados, incluindo os seguintes:

1. Todos os pesquisadores participarão de uma sessão de treinamento antes do início do estudo para garantir a consistência dos procedimentos do estudo, incluindo a coleta de dados.
2. Os pesquisadores poderão telefonar e/ou participar de breves reuniões *on-line* com o centro de coordenação do estudo para resolver questões ou problemas que possam surgir, bem como participar de grupos de discussão por meio de mensagens eletrônicas.
3. As verificações de dados para identificar inconsistências serão realizadas periodicamente. Os centros serão notificados sobre as inconsistências para que façam as correções.
4. O centro de coordenação analisará relatórios mensais detalhados sobre triagem, inclusão, seguimento, consistência e integridade dos dados e tomará medidas imediatamente para resolver quaisquer problemas.
5. O monitoramento do local será realizado durante o estudo para uma amostra dos locais. Esse monitoramento será realizado remotamente.

### Análise estatística

Um plano detalhado de análise estatística pode ser encontrado no Apêndice 2 do Material Suplementar. As características resumidas do plano de análise estatística estão descritas a seguir. Todas as análises irão aderir ao princípio de uma abordagem modificada de intenção de tratar. Podem ocorrer exclusões de participantes após a randomização por recusas, dada a natureza retrospectiva (*opt-out*) do consentimento informado. Avaliaremos o efeito da estratégia STAMINA *versus* a estratégia ARDSNet no desfecho primário usando um modelo ordinal randômico, com ajuste para idade basal, diagnóstico da COVID-19, taxa ventilatória basal e PEEP. Os resultados serão relatados como razão de chance proporcional (ORp - *proportional odds ratio*) com intervalos de confiança de 95%. Para desfechos secundários binários, compararemos as razões de chances e os intervalos de confiança de 95% ajustados para as mesmas variáveis e usaremos interceptações aleatórias no centro. Para resultados contínuos, apresentaremos a diferença média e o intervalo de confiança de 95%.

Analisaremos o efeito das intervenções nos seguintes subgrupos de interesse:

1. Pacientes com e sem diagnóstico da COVID-19 (confirmado por reação em cadeia da polimerase - PCR);
2. Pacientes com DP acima ou abaixo de 15cmH_2_O antes da randomização.

### Monitoramento de dados

O monitoramento de dados em cada centro envolvido neste estudo clínico será realizado por uma equipe de gestão de centros altamente treinada e qualificada. Ela será responsável pela realização de visitas planejadas ao local para garantir a precisão, a integridade e a conformidade dos dados com o protocolo do estudo e os requisitos regulatórios. Além disso, a equipe analisará meticulosamente os documentos de origem, formulários de relato de caso e outros documentos relacionados ao estudo para verificar a integridade e a qualidade dos dados coletados.

### Eventos adversos

Coletaremos e analisaremos dados sobre suspeitas de eventos adversos graves (EAGs) inesperados. Esses EAGs devem ser relatados ao centro coordenador dentro de 24 horas. Quaisquer EAGs devem ser relatados ao centro de coordenação dentro de 24 horas. Todos os EAGs serão manejados imediatamente pela equipe de tratamento e julgados pela equipe do centro coordenador. Eventos inesperados serão relatados à equipe de pesquisa dentro de 24 horas. Prevê-se que a população de pacientes na UTI apresentará uma série de aberrações nos valores laboratoriais, sinais e sintomas, devido à gravidade da doença subjacente e ao impacto dos tratamentos padrão na UTI. Esses eventos não constituirão necessariamente eventos adversos ou EAGs, salvo se forem considerados relacionados ao tratamento do estudo ou, no julgamento clínico do investigador principal, não forem eventos reconhecidos como consistentes com a doença crítica e/ou doenças crônicas subjacentes dos participantes e com o curso clínico esperado. O principal EA de interesse é o barotrauma (pneumotórax, pneumomediastino e enfisema subcutâneo).

### Auditoria

A auditoria será realizada pela equipe operacional do patrocinador para ajudar a manter a objetividade e a credibilidade na avaliação da conformidade do estudo com o protocolo, os requisitos regulatórios e as diretrizes de Boas Práticas Clínicas (BPC).

### Danos

Considerando que o paciente receberia suporte de VM mesmo se não estivesse participando do estudo, é de se esperar um aumento mínimo do risco já inerente ao contexto da VM, que inclui a necessidade de sedação, queda da pressão arterial (hipotensão), aumento do risco de pneumonia e uso de agentes bloqueadores neuromusculares.

### Violações e desvios do protocolo

Os principais desvios relacionados aos critérios de inclusão, aos critérios de exclusão, à condução do estudo e ao manejo do paciente devem ser relatados e monitorados pelo centro coordenador.

### Organização e supervisão do estudo

O comitê diretivo do estudo tem 14 membros e é responsável pelo desenvolvimento do protocolo do estudo, pela elaboração do manuscrito e pelo envio dele para publicação (Apêndice 1 - Material Suplementar). Uma equipe do *Hcor Research Institute* está coordenando o estudo em associação com a BRICNet. O *Hcor Research Institute* é responsável pela condução do estudo e pelo tratamento e controle da qualidade dos dados.

O comitê de monitoramento de dados (DMC - *data monitoring committee*) é composto de um estatístico externo e dois pesquisadores, todos especialistas em medicina intensiva (consulte o Estatuto do DMC no Apêndice 3 - Material Suplementar). O DMC é responsável pela análise provisória e por fornecer orientação ao comitê diretivo em relação à continuação e à segurança do estudo após as análises provisórias.

### Situação atual do estudo

A aprovação ética foi obtida em 15 de julho de 2021. Desde então, 207 pacientes foram alocados aleatoriamente para um dos braços do estudo. O primeiro paciente foi incluído no estudo em 4 de setembro de 2021. A primeira reunião do DMC foi realizada em 11 de abril de 2022. Após analisar os dados de acordo com o Estatuto do DMC (Apêndice 3 - Material Suplementar), o DMC recomendou a continuação do estudo conforme planejado no protocolo.

### Considerações éticas e divulgação

O estudo foi elaborado de acordo com as diretrizes de BPC e os princípios da Declaração de Helsinque. Este estudo foi aprovado pelo Comitê de Ética em Pesquisa (CEP) do Hcor em 15 de julho de 2021 (aprovação 4.848.945) e pelo CEP de cada centro participante. O consentimento será solicitado a todos os representantes legais ou familiares. Durante o processo de obtenção do Termo de Consentimento Livre e Esclarecido (TCLE), os pesquisadores do estudo STAMINA tentarão obter o TCLE antes da inclusão do paciente, sempre que possível. No entanto, acreditamos que, em vários casos, a obtenção do TCLE após a randomização seria totalmente justificável, dada a urgência da decisão de tratamento. Este estudo foi aprovado pelo CEP. Portanto, se não for possível entrar em contato com o representante legal antes da inclusão no estudo, o TCLE será obtido após a inclusão, dentro de 48 horas, por meio de seu familiar/representante legal. Deve-se priorizar o TCLE em papel. Entretanto, considerando as restrições de visitas de familiares às instalações hospitalares, especialmente durante o período de pandemia da COVID-19, alternativas digitais, como *e-mail* ou ligações telefônicas gravadas, podem ter sido empregadas. Se o paciente morrer antes de o pesquisador entrar em contato com o representante legal, o CEP do centro será informado sobre as permissões de uso dos dados. O estudo será enviado para publicação após a conclusão, independentemente dos resultados. A preparação do manuscrito será uma responsabilidade inalienável do comitê diretivo. O artigo principal será de autoria dos membros do comitê diretivo e dos pesquisadores dos principais locais de recrutamento, que podem contribuir intelectualmente para o manuscrito.

### Papel do patrocinador

O Ministério da Saúde do Brasil, por meio de seu Programa de Apoio ao Desenvolvimento Institucional do Sistema Único de Saúde (Proadi-SUS), concede financiamento para a STAMINA. O patrocinador não está envolvido no desenho do estudo, na análise dos dados, na preparação do manuscrito e nem na decisão de enviar os resultados para publicação.

### Declaração de interesse

Os membros do Comitê Diretivo declaram não haver declarações de interesse.

## DISCUSSÃO

O estudo STAMINA foi planejado como um ensaio clínico randomizado, multicêntrico e controlado com o objetivo de comparar duas estratégias ventilatórias em pacientes com SDRA devido à PAC. A estratégia STAMINA (grupo intervenção) consiste em limitar a DP ao otimizar a PEEP guiada pela melhor complacência do sistema respiratório e reduzir o volume corrente se a DP permanecer > 14cmH_2_O. No grupo ARDSnet, a titulação da PEEP será realizada usando a tabela de PEEP baixa-FiO_2_. Nosso objetivo é avaliar se essa estratégia de limitação da DP reduzirá o número de dias sem VM em comparação com o grupo controle. Uma estratégia de DP limitada já foi estudada em um estudo de viabilidade.^(14)^ No grupo com DP limitada, foi usado um volume corrente muito baixo para diminuir a DP. As principais preocupações de segurança foram a hipercapnia e a acidose respiratória. Entretanto, o aumento da frequência respiratória foi suficiente para evitar a acidose respiratória grave na maioria dos pacientes. Não foram levantadas questões de segurança. A melhor estratégia de titulação da PEEP em pacientes com SDRA permanece controversa.^(11^^,^^15^^,^^16)^ A estratégia com maior validação na literatura continua sendo a tabela PEEP-FiO_2_, que foi construída para correlacionar os valores de PEEP conforme as necessidades de FiO_2_.^(8^^,^^9)^ Nenhuma estratégia demonstrou ser superior à tabela PEEP-FiO_2_ em ensaios clínicos randomizados robustos.^(17)^ O ensaio ART foi conduzido para estudar o recrutamento alveolar com pressões elevadas, seguido da titulação da PEEP para melhor adesão em pacientes com SDRA. Essa estratégia resultou em aumento da mortalidade, principalmente devido à ocorrência de barotrauma.^(1)^ No entanto, os estudos LOVS e EXPRESS foram realizados para testar estratégias alternativas à tabela PEEP-FiO_2_; porém, seus resultados não mostraram superioridade.^(18^^,^^19)^ Até o momento, não foram realizadas estratégias ventilatórias que visem à DP como meta e desfechos centrados no paciente em estudos controlados e randomizados bem desenhados.^(20^^,^^21)^ O estudo STAMINA pode mostrar se essa estratégia é superior ao padrão atual de tratamento (tabela PEEP). Se nosso estudo mostrar o benefício de limitar a DP otimizando a PEEP guiada pela complacência do sistema respiratório e diminuir o volume corrente aumentando o número de dias sem ventilação, poderemos contribuir para mudanças na prática clínica e melhorias no tratamento da SDRA.

## Funções e responsabilidades:

Material suplementar (Apêndice 1)
